# Historical introgression as a driver of diversification of diploid *Picris* (Compositae) in the Mediterranean Basin

**DOI:** 10.1111/tpj.70565

**Published:** 2025-11-11

**Authors:** Juan Manuel Gorospe, Tomáš Fér, Ivan Rurik, Peter Vďačný, Jaromír Kučera, Ali A. Dönmez, İbrahim Sırrı Yüzbaşıoğlu, Zübeyde Uğurlu Aydın, Magdalena Lučanová, Roswitha Elisabeth Schmickl, Marek Slovák

**Affiliations:** ^1^ Department of Botany, Faculty of Science Charles University Prague 128 01 Czechia; ^2^ Department of Evolutionary Plant Biology Institute of Botany of the Czech Academy of Sciences Průhonice 252 43 Czechia; ^3^ Department of Zoology Comenius University Bratislava Bratislava 842 15 Slovakia; ^4^ Institute of Botany, Plant Science and Biodiversity Centre Slovak Academy of Sciences Bratislava 845 23 Slovakia; ^5^ Department of Biology, Faculty of Science, Molecular Plant Systematic Laboratory (MOBIS) Hacettepe University Ankara Turkey; ^6^ Department of Botany, Faculty of Science İstanbul University İstanbul Turkey; ^7^ Department of Botany, Faculty of Science University of South Bohemia České Budějovice 370 05 Czechia

**Keywords:** diploid hybridization, endemism, historical introgression, Mediterranean, *Picris*, trait evolution

## Abstract

The Mediterranean Basin is recognized as one of the world's most prominent biodiversity hotspots, where past climatic changes have driven range shifts, secondary contact between populations, and gene exchange. This study investigates the impact of historical introgression on the diversification of diploid members of the genus *Picris* (Compositae). Using nuclear and plastid genome data obtained through the Hyb‐Seq approach, we assess whether introgression contributed to the evolution of the Mediterranean *Picris*, potentially giving rise to multiple regional endemics. We also test whether introgression was associated with the transfer of traits such as life strategy and fruit morphology, which are involved in habitat‐specific adaptation. Phylogenetic network analysis revealed two major introgression events that shaped evolutionary trajectories within the genus. The earliest and most complex events involved the Turkish endemic *P. campylocarpa*, which hybridized with the most recent common ancestor (MRCA) of the *P. cyprica*–*P. pauciflora* lineage and with the MRCA of the B1 subclade, comprising the *P. hieracioides* group and the *P. scaberrima*–*P. strigosa* lineage. The latter introgression preceded shifts from iteroparity to semelparity and from heterocarpy to homocarpy, ruling out an adaptive introgression origin for these traits. Nevertheless, all detected historical introgression events contributed to the diversification of diploid *Picris* taxa.

## Significance Statement

The remarkable biodiversity of the Mediterranean Basin has developed over extensive geological timescales predominantly through geographical and ecological divergence, frequently associated with polyploidy. Recurrent paleoclimatic and paleogeological oscillations, however, have repeatedly facilitated the formation of contact zones, where hybridization has played a key evolutionary role in driving diversification and speciation, thereby underpinning the exceptional biotic richness within this global biodiversity hotspot.

## INTRODUCTION

The Mediterranean Basin is the richest of the five Mediterranean floristic climatic zones in terms of both species number and territorial extent (Cowling et al., 1996; Vargas, 2020), and it ranks as the third largest biodiversity hotspot in the world (Lopez‐Alvarado & Farris, 2022). Hosting up to 25 000 vascular plant species (Greuter, 1991), its diversity is characterized not only by a wide array of genera and families (Vargas, 2020) but also by a broad range of functional groups (Tavşanoğlu & Pausas, 2018). Substantial floristic changes, particularly during the Neogene, were driven by climatic cooling and aridification. These changes led to the extinction of many plant lineages and entire plant communities (Postigo Mijarra et al., 2009), while simultaneously fostering speciation processes (Vargas, 2020).

A review of 27 angiosperm clades by Vargas et al. (2018) suggested that allopatric speciation is the predominant mode of speciation in the Mediterranean Basin. The region's complex geomorphology, including islands, peninsulas, and mountain ranges, promotes geographical isolation, which limits gene flow, encourages divergent adaptation, and facilitates the establishment of reproductive barriers (Vargas et al., 2018). More than half of all plant species in the Mediterranean Basin are endemic (Thompson, 2020; Thompson et al., 2005), often restricted to islands or mid‐ to high‐altitude mountains (Fernández‐Mazuecos et al., 2014; Jiménez‐Mejías et al., 2015; Lavergne et al., 2004). Although direct evidence for ecological speciation, where environmental differences drive lineage divergence and reproductive isolation through selection (Rundle & Nosil, 2005; Schluter, 2009), is limited, ecological differences among closely related species suggest that it may also play a significant role in the plant diversity of the Mediterranean Basin (Fernández‐Mazuecos & Vargas, 2010; Thompson, 2020).

Following the establishment of the Mediterranean climate in the Pliocene (~3.2 million years ago [Mya]; Suc, 1984), the Quaternary glacial–interglacial cycles brought significant climatic fluctuations to the Mediterranean Basin. These cycles led to both the extinction and retreat of warm‐adapted lineages into glacial refugia during colder periods, followed by range expansions during warmer phases (Médail & Diadema, 2009). These contraction‐expansion dynamics created transient contact zones between previously isolated populations, facilitating hybridization and introgression (Nieto Feliner, 2014; Thompson, 2020). Occasionally, introgression introduces novel genetic variation, including traits of adaptive value. Adaptive introgression refers to the transfer of genomic regions from a donor species that confer a fitness advantage to the recipient species (Suárez‐González et al., 2018). It may be more common among polyploids due to reduced reproductive barriers and the genomic effects of whole‐genome duplication (Schmickl & Yant, 2021). Nonetheless, cases of adaptive introgression between diploids are well documented in both plants and animals (Edelman & Mallet, 2021; Schmickl et al., 2017). Introgression has been implicated in reverse speciation, such as between endemic alpine plants and widespread lowland congeners (Gómez et al., 2015; Slovák et al., 2023), but it has also been shown to promote diversification and evolutionary radiations (Hudson et al., 2011; Joyce et al., 2011; Kozak et al., 2021; Meier et al., 2017; Qian et al., 2023; Skopalíková et al., 2023; Stankowski & Streisfeld, 2015; Svardal et al., 2020).

Despite extensive literature on plant evolution and systematics documenting hybridization across numerous Mediterranean taxa, the impact of historical introgression on the diversification of the region's flora remains largely unexplored (but see Agudo et al., 2023; Marques et al., 2018; Nieto Feliner et al., 2023; Tiburtini et al., 2025). While this knowledge gap may soon narrow due to the increased data resolution of common high‐throughput genomic technologies, most existing studies have focused on hybridization associated with polyploidy and the formation of allopolyploids (Kantor et al., 2023; Skubic et al., 2023; Šlenker et al., 2021; Valdés‐Florido et al., 2024) or on gene flow between dysploid and euploid cytotypes (Liu et al., 2022). Although cases of homoploid diploid hybridization have also been reported (Albaladejo & Aparicio, 2007; Blanco‐Pastor et al., 2012; Lakušić et al., 2024; Radosavljević et al., 2019), the role of historical introgression at the diploid level in shaping plant diversification in the Mediterranean Basin remains elusive and poorly understood. Recent hybridization between diploids is often evident from morphological and ecological traits alone, as demonstrated by foundational studies (e.g., Anderson, 1953; Grant, 1981; Stebbins, 1959) and more recent examples from Mediterranean plants (Gristina et al., 2014; Lakušić et al., 2024; Peruzzi et al., 2011; Radosavljević et al., 2019). A well‐documented case is the extensive introgression among diploid oak species, resulting in distinct morphological and ecological types (Dodd & Afzal‐Rafii, 2004; McVay et al., 2017). Outside the Mediterranean, hybridization among diploid *Helianthus* species represents such a case, as it produced extreme phenotypes and ecotypes (Rosenthal et al., 2002).

Members of the genus *Picris* (Compositae) are well suited for studying the effect of historical hybridization on adaptive introgression and the diversification of diploid taxa in the Mediterranean Basin (Lack, 1974; Slovák et al., 2012, 2014, 2018; Smalla, 2000). The center of diversification for this genus, comprising approximately 50 taxa, coincides with its highest diversity in the Mediterranean region (Lack, 1974; Slovák et al., 2018). Two major lineages, Clade A and Clade B, diverged shortly after the genus originated in the Pliocene, with most species emerging during the Quaternary (Slovák et al., 2018). Clade A primarily occupies North Africa and extends into eastern and tropical Africa, the Arabian Peninsula, and the Iberian Peninsula, while Clade B is distributed in the northeastern Mediterranean (Figure S1). The latter also includes the *P. hieracioides* group, which comprises multiple closely related taxa distributed across temperate Eurasia and Australia (Slovák et al., 2018). Strong incongruence between nuclear and plastid phylogenies, along with the presence of basal polytomies, suggests both historical and contemporary hybridization events. However, particularly within Clade B, the low informativeness of previously used genetic markers as well as incomplete lineage sorting (ILS) may also contribute to these patterns (Slovák et al., 2014, 2018). All *Picris* taxa, except one, are diploid with a chromosome number of 2*n* = 2*x* = 10 (Astuti et al., 2015; Holzapfel, 1994; Lack, 1974; Slovák et al., 2007, 2018; Smalla, 2000). *Picris* species exhibit variation in key functional traits such as longevity (life strategy), including semelparous taxa, which are annuals or biennials that reproduce only once, and iteroparous taxa, which are mostly short‐lived perennials that produce seeds multiple times during their lifespan (Friedman & Rubin, 2015). They also vary with respect to fruit morphology: they can be heterocarpic or homocarpic. Capitula of homocarpic species bear morphologically and functionally uniform cypselas with plumose pappi, which are wind‐dispersed. Heterocarpic taxa, in contrast, produce dimorphic cypselas: central cypselas with pappi that are dispersed, and peripheral cypselas lacking pappi, remaining attached to the receptacle in close proximity to the mother plant (Baskin & Baskin, 2014; Cruz‐Mazo et al., 2009; Imbert, 2002). Both of these life‐history traits may influence environmental adaptation and diversification and are thus considered crucial for the evolution and taxonomy of the genus (cf. Lack, 1974; Slovák et al., 2018). *Picris* species commonly colonize open, sunny habitats such as dunes, steppes, deserts, and semi‐deserts, as well as arid to mesophilic or even high‐alpine grasslands and rocky habitats (Table S1; Holzapfel, 1994; Lack, 1974; Slovák et al., 2012, 2014, 2018; Smalla, 2000). It has been shown that longevity in *Picris* species is correlated with the evolution of fruit morphology and functionality, traits likely shaped by environmental pressures. Semelparous species tend to thrive in arid, unpredictable habitats, while iteroparous species are more common in cooler, more stable environments (Lack, 1974, 1979; Slovák et al., 2012, 2014, 2018). Interestingly, the combination of homocarpy and iteroparity along with their association with predictable habitats is characteristic primarily of the largest species group within the genus, the *P. hieracioides* group. Thus, it has been hypothesized that this trait combination might accelerate diversification and facilitate rapid transcontinental colonization, particularly in the *P. hieracioides* group (Slovák et al., 2018; see also Drummond et al., 2012 for a study on the genus *Lupinus*).

Here, we hypothesize that historical introgression events have influenced the diversification and speciation of diploid *Picris* within Clade B in the Mediterranean Basin. To test this hypothesis, we assessed whether historical introgression occurred multiple times among different subclades and lineages of Clade B. Using phylogenetic network‐based trait reconstruction, we further tested whether introgression promoted the transfer of longevity and fruit traits, and whether such transfer coincided with the colonization of specific environments.

## RESULTS

### Nuclear and plastome phylogenies

The nuclear phylogeny inferred from dataset A (89 individuals and 981 concatenated nuclear loci) using RAxML‐NG confirmed the monophyly of the genus *Picri*s with full statistical support (bootstrap support, BS = 100%). Two major clades, Clade A and Clade B sensu Slovák et al. (2018), were both strongly supported (BS = 100%). Clade A comprised all African taxa along with some species from the Iberian and Arabian Peninsulas, while Clade B included Eurasian taxa (Figure S1). Within Clade B, two major subclades, B1 and B2 sensu Slovák et al. (2018), also received full support (BS = 100%; Figure S2). Subclade B1 included homocarpic, predominantly iteroparous species from the *P. hieracioides* group, along with *P. strigosa* M.Bieb. and *P. scaberrima* Guss. ex Ten. (hereafter referred to as the *P. hieracioides* clade) which are distributed from Europe across Asia to eastern and southern Australia. Subclade B2, also well supported, comprised semelparous species, both homocarpic and heterocarpic, mostly from the northeastern Mediterranean.

**Figure 1:**
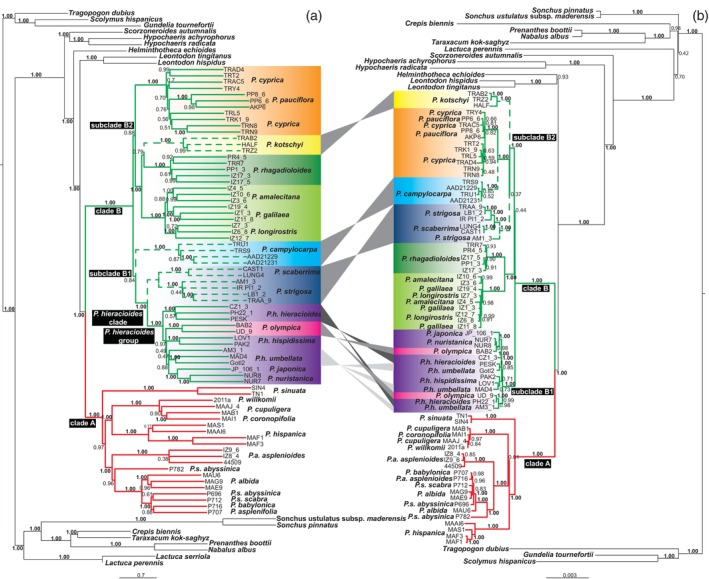
Nuclear and plastome phylogenies. (a) Coalescent‐based species tree inferred using ASTRAL from 981 exon trees and 89 individuals of 72 *Picris* and 17 outgroup taxa. Branch support is indicated by local posterior probability values. (b) Maximum likelihood tree generated with RAxML‐NG from concatenated plastome data, comprising 119 coding and non‐coding loci across 88 *Picris* and outgroup accessions. Bootstrap support values are shown above branches. Taxa exhibiting discordant placements between the nuclear (ASTRAL) and plastome (RAxML‐NG) trees are marked with dashed lines and connected by gray lines between the trees. In the case of subspecies names of *P. hieracioides*, the species name is shortened (h.).

The ASTRAL coalescent‐based species tree also supported the division of *Picris* into Clades A and B with full statistical support (local posterior probability, LPP = 1.0; Figure 1a). Subclades B1 and B2 showed the same taxon composition as in the RAxML‐NG phylogeny, though with lower support (LPP = 0.84 and 0.88, respectively). However, the placement of *P. campylocarpa* Boiss. & Heldr. from Türkiye differed between the RAxML‐NG (Figure S2) and ASTRAL trees (Figure 1a). In the RAxML‐NG tree, it was placed in subclade B2, sister to *P. cyprica* Lack and *P. pauciflora* Willd. (BS = 92%), whereas in the ASTRAL tree it appeared in subclade B1, sister to the remaining taxa (LPP = 0.84). In both phylogenies, most species and subspecies formed well‐supported, taxon‐specific groups. Coalescent‐based analyses using SNAPP confirmed the presence of subclades B1 and B2 (Figure S3), but placed the lineage comprising *P. campylocarpa*, *P. cyprica*, and *P. pauciflora* (posterior probability, PP = 0.97) within subclade B1 (PP = 1.0), contrasting with the RAxML‐NG and ASTRAL topologies. PhyParts analysis revealed strong gene/species tree discordance, as only a few genes supported the species tree topology (Figure S4).

The plastome phylogeny also recovered the two major Clades A and B with full statistical support (Figure 1b). However, several taxon‐specific clades showed discordant patterns compared to the nuclear phylogeny, notably *P. campylocarpa*, *P. kotschyi* Boiss., *P. strigosa*, and *P. scaberrima* (Figure 1a,b; Figures S2 and S3). Additionally, taxa within the *P. hieracioides* group appeared non‐monophyletic in the plastome phylogeny, in contrast to the nuclear analyses (Figure 1a,b).

The crown divergence of Clade B was estimated to 5.04 Mya (highest posterior density [HPD]: 5.00–5.08 Mya). Subclades B1 and B2 began diversifying 3.87 Mya (HPD: 3.65–4.12 Mya) and 4.69 Mya (HPD: 4.61–4.75 Mya), respectively. Most taxa within Clade B originated during the Quaternary, which was also the onset of their intraspecific diversification (Figure S5).

### Network and genetic admixture analyses

The Neighbor‐Net analysis revealed a complex reticulation pattern, with the primary conflict in the backbone of the network involving *P. campylocarpa*, *P. cyprica*, *P. pauciflora*, and subclade B1. Additional conflicting splits connected *P. kotschyi* with *P. rhagadioloides* (L.) Desf. (Figure S6).

**Figure 2:**
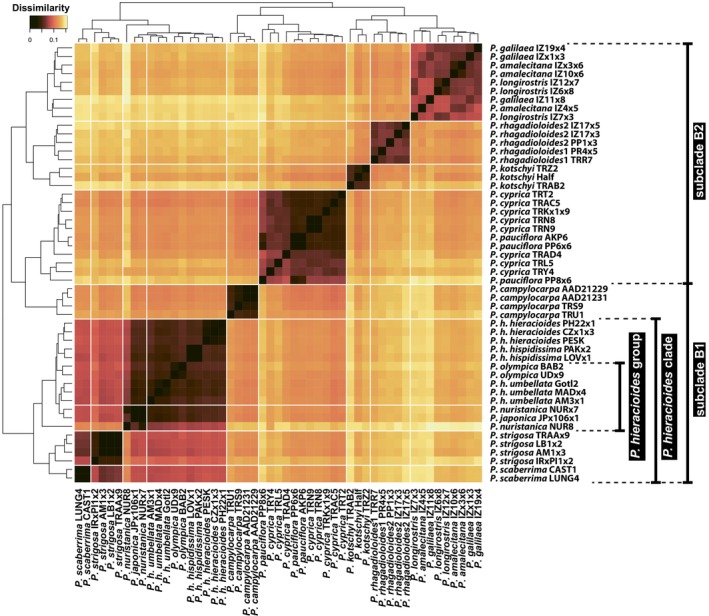
Heatmap of pairwise relatedness among 52 individuals belonging to 17 *Picris* taxa, and 23 608 single nucleotide polymorphisms.

A similarity heatmap based on 52 individuals and 23 608 SNPs indicated the presence of both single‐ and multispecies lineages. *Picris campylocarpa* appeared to share ancestry with both the *P. hieracioides* clade and the *P. cyprica–P. pauciflora* lineage. The most pronounced signs of genetic admixture were observed among members of the *P. hieracioides* clade although weaker signals were also detected in all other East Mediterranean taxa (Figure 2).

**Figure 3:**
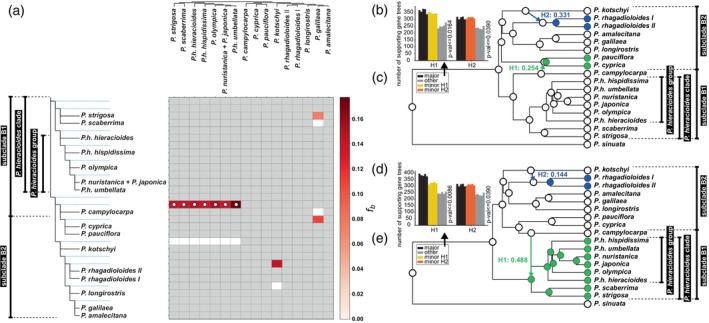
Network and genetic admixture analyses. (a) Results of *f*‐branch statistic based on 52 individuals and 2390 single nucleotide polymorphisms, inferred using Dsuite with a significance threshold of *P* value ≤0.001 (marked with white dots) and *P* value ≤0.05 (no dots). Gray shading indicates taxon combinations for which the *f*‐branch statistic could not be computed due to sampling limitations or incompatibility with the phylogenetic tree topology. (b, d) Results of TWISST analyses for the prevailing and alternative network topologies. (c, e) Prevailing and alternative network topologies of *Picris* Clade B as estimated by SNaQ. *γ* values are shown above the introgression edges. Colors indicate taxa affected by introgression events.

The *f*‐branch statistic using Dsuite detected a single introgression event with strong statistical support (*P* ≤ 0.001), occurring between ancestors of the entire clade including *P. campylocarpa*, *P. cyprica*, and *P. pauciflora*, and the entire *P. hieracioides* clade (Figure 3a). At a lower significance threshold (*P* ≤ 0.05), additional introgression signals were found between the ancestor of the *P. rhagadioloides* lineage and *P. kotschyi*, as well as weak signals between *P. galilaea* (Boiss.) Eig and *P. strigosa*, and between *P. galilaea* and the ancestor of the *P. cyprica–P. pauciflora* lineage (Figure 3a).

Two SNaQ networks, each containing two introgression events, were selected based on the criterion of having the highest number of reticulations supported by strong or moderate support across five specimen resamplings (networks with more than two reticulations yielded inconsistent results across different resampling runs). These networks differed only in the first introgression event, while the second was identical in both. Regarding the first introgression event, *P. campylocarpa* was sister to subclade B1 and introgressed into the most recent common ancestor (MRCA) of the *P. cyprica–P. pauciflora* lineage (*γ* = 0.254; Figure 3c). This scenario was strongly supported by three resamplings and weakly by a fourth. In the alternative network, *P. campylocarpa* was sister to the *P. cyprica–P. pauciflora* lineage and introgressed into the MRCA of subclade B1 (*γ* = 0.488; Figure 3e), with strong support from one resampling, moderate support from another, and weak support from a third. The second, shared introgression event involved gene flow from the Near East endemic *P. kotschyi* into the northern Mediterranean widespread *P. rhagadioloides* lineage (*γ* = 0.331 in the prevailing network and *γ* = 0.144 in the alternative network).

Both introgression events were tested using TWISST (Figure 3b,d). The null hypothesis that these patterns were caused by ILS was rejected across all five specimen resamplings. Specifically, the ILS scenario was refuted for the first introgression event at *P* ≤ 0.0164 (prevailing network) and *P* ≤ 0.0086 (alternative network), and for the second introgression event at *P* ≤ 0.039 in both networks.

### Ancestral state reconstruction of three traits

**Figure 4:**
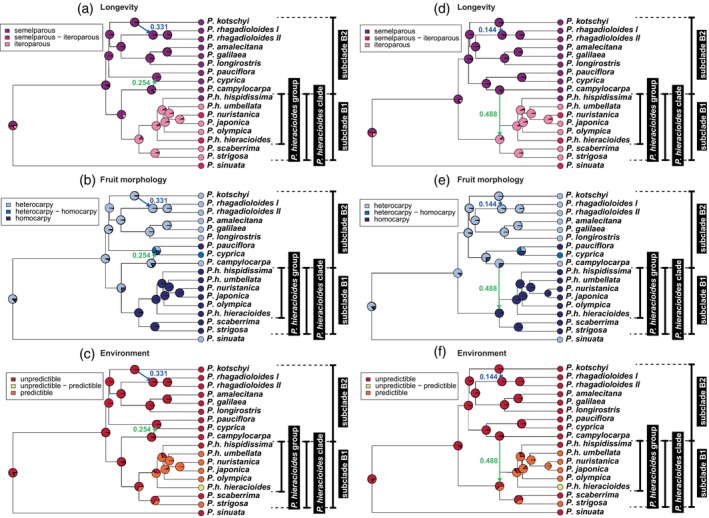
Ancestral state reconstruction of two intrinsic and one extrinsic traits in *Picris*. (a–c) Reconstructions based on ultrametrized prevailing and (d–f) alternative SNaQ networks, performed under the best‐fit equal rates model using PhyloNetworks. Gene trees from the ASTRAL phylogeny, based on 18 *Picris* taxa and 964 nuclear loci, were used as scaffolds for SNaQ network construction.

The evolution of three traits (longevity, fruit morphology, environmental predictability) was reconstructed on both favored SNaQ networks using the equal rates substitution model. Across all analyses, the MRCA of *Picris* Clade B was most plausibly a semelparous, heterocarpic herb adapted to unpredictable habitats (Figure 4a–c for the prevailing network and Figure 4d–f for the alternative network).

Semelparity evolved independently once within subclade B1, specifically in *P. hieracioides* subsp. *hispidissima*, while transitional stages between iteroparity and semelparity appeared twice, in *P. nuristanica* and *P. hieracioides* subsp. *hieracioides*. The reconstruction of the life strategy for the MRCA of subclade B1 depended on the phylogenetic position of *P. campylocarpa* (Figure 4a,d). In the alternative scenario, where introgression occurred between the semelparous *P. campylocarpa* and the iteroparous MRCA of subclade B1, semelparity was not directly introgressed into the ancestors of the *P. hieracioides* clade (Figure 4d).

Fruit morphology evolved in a pattern similar to that of longevity. Homocarpy emerged within Clade B at least twice: once at the base of the *P. hieracioides* clade and once in *P. pauciflora* (Figure 4b,e). In the alternative network, heterocarpy was not introgressed from *P. campylocarpa* into the ancestor of the *P. hieracioides* clade (Figure 4e). Adaptation to predictable environments evolved recurrently in several taxa within subclade B1 (Figure 4c,f).

## DISCUSSION

### Complex historical introgression events shaped the intrageneric evolution of *Picris*

The integration of nuclear and plastid genome data revealed several historical introgression events among diploid members of *Picris* Clade B, which have influenced their evolutionary trajectories and speciation patterns. These events were difficult to detect in earlier studies, despite being suggested by other biological traits, because the involved *Picris* taxa are morphologically similar and share the same chromosome number and diploid status (Lack, 1974; Slovák et al., 2012; Slovák, Urfus, et al., 2009; Slovák, Vít, et al., 2009). The most compelling indicator of historical introgression is the pronounced and well‐supported cytonuclear discordance (cf. Cai et al., 2021; García et al., 2017; Morales‐Briones et al., 2021; Rose et al., 2021; Slovák et al., 2023; Sun et al., 2015). The combination of basal polytomies and incongruences in the nuclear and plastome phylogenies (Figure 1a,b; Figures S2 and S3), central conflicting splits in the Neighbor‐Net network (Figure S6), and strong gene/species tree incongruence (Figure S4) suggests that the shallow basal structure of Clade B is linked to rapid diversification accompanied by hybridization and/or ILS. Using multiple statistical approaches (Figures 2 and 3a–e), we identified two major introgression events. The first is complex and not easily interpreted, as it involves different ancestral lineages that acquired genetic material from the eastern Mediterranean endemic *P. campylocarpa*. In one scenario, *P. campylocarpa* introgressed into the MRCA of the eastern Mediterranean *P. cyprica–P. pauciflora* lineage (Figures 2 and 3b,c). In the alternative scenario, introgression occurred into the MRCA of subclade B1 (Figures 2 and 3d,e). The second introgression event involved the eastern Mediterranean endemic *P. kotschyi*, which contributed genetic material to the MRCA of the *P. rhagadioloides* lineage. This event is consistently supported by TWISST, SNaQ, and *f*‐branch analyses (Figure 3a–e). Additional introgression events are likely among closely related taxa within the *P. hieracioides* group (Slovák et al., 2012, 2014, 2018) and within the Near East lineage comprising *P. galilaea*, *P. amalecitana* (Boiss.) Eig, and *P. longirostris* Sch.Bip. (Figure 2; Figure S6).

Detecting historical introgression is notoriously challenging, especially when multiple events occur at different time horizons (e.g., Cai et al., 2021; Kremer & Hipp, 2020; Slovák et al., 2023). The use of Hyb‐Seq data in this study, representing a relatively small portion of the genome, combined with the sensitivity of detection methods to missing data (e.g., phylogenetic networks, *f*‐branch statistic), may explain why the number of inferred introgression events varied across approaches (cf. Kandziora et al., 2022). The first introgression event involving *P. campylocarpa* yielded two plausible scenarios, each supported by different subsets of data and analytical approaches. These alternatives may partly reflect methodological limitations. Notably, SNaQ cannot infer introgression between sister lineages (Solís‐Lemus et al., 2017), which limits its ability to detect both introgression events involving *P. campylocarpa* within a single topology. As a result, this event was represented by two alternative placements of *P. campylocarpa* in the phylogenetic networks: either sister to subclade B1 (Figure 3c) or to the *P. cyprica–P. pauciflora* lineage within subclade B2 (Figure 3e). From a biological perspective, this introgression likely reflects an ancient event involving at least one parental taxon that contributed to the ancestry of modern lineages (see Stull et al., 2023 for review). Consequently, the introgression signal may have weakened over time. Ancestral area reconstructions from our previous study (Slovák et al., 2018) suggest that *P. campylocarpa*, along with the ancestors of subclade B1 and the *P. cyprica–P. pauciflora* lineage, likely originated in West Asia. Notably, some populations of *P. cyprica* are currently sympatric or parapatric with *P. campylocarpa* (Lack, 1974; personal observations by M. Slovák and J. Kučera). Given this long‐term geographical overlap, it is highly plausible that *P. campylocarpa* hybridized with both MRCAs, facilitating genetic exchange and influencing the evolutionary trajectories of these lineages. In light of this, we interpret both scenarios for the first introgression event as biologically credible and complementary rather than mutually exclusive.

### Historical introgression modulated evolutionary trajectories and speciation patterns in the Mediterranean *Picris*

The remarkable plant diversity of the Mediterranean Basin has been shaped not only by geographical and ecological divergence and polyploidy but also by historical and recent hybridization (Marques et al., 2018). Approximately 5.6% of Mediterranean plant species are of hybridogenous origin (Marques et al., 2018), although this figure is likely underestimated due to limited data, particularly from the eastern Mediterranean. Consequently, the extent to which hybridization contributes to diversification and evolutionary radiations in the Mediterranean Basin remains largely untested (but see Yang et al., 2021). More broadly, historical hybridization and introgression have been shown to stimulate diversification across a wide range of organisms (Hudson et al., 2011; Joyce et al., 2011; Kozak et al., 2021; Meier et al., 2017; Qian et al., 2023; Skopalíková et al., 2023; Stankowski & Streisfeld, 2015; Svardal et al., 2020). In this study, we suggest that the first complex introgression event, between *P. campylocarpa* and the MRCA of the *P. cyprica–P. pauciflora* lineage and the MRCA of subclade B1, contributed to the emergence of several Mediterranean endemics, including *P. cyprica*, *P. pauciflora*, *P. hieracioides* subsp. *hispidissima*, *P. olympica*, and *P. scaberrima* from subclade B1 (Astuti et al., 2015; Lack, 1974; Slovák et al., 2014, 2018; Slovák, Urfus, et al., 2009; Slovák, Vít, et al., 2009). The second introgression event, which occurred between the Mediterranean endemics *P. kotschyi* and *P. rhagadioloides*, likely contributed to the diversification of two morphologically similar lineages within the latter taxon.

A comparison of the two subclades affected by the first historical introgression event revealed differences in diversification intensity. While subclade B1 underwent extensive diversification, the *P. cyprica–P. pauciflora* lineage remained species‐poor, comprising only two taxa. However, the high intraspecific genetic variability observed in *P. cyprica* suggests ongoing diversification or the presence of cryptic taxa (Figure 1a,b; Figures S2, S4, and S5; Slovák et al., 2018). This is particularly notable given its restricted distribution along the southern Turkish and Cypriot coasts (Lack, 1974). We propose that, although not as speciose as subclade B1, *P. cyprica* is undergoing fine‐scale diversification likely facilitated by gene flow between the MRCA of the *P. cyprica–P. pauciflora* lineage and *P. campylocarpa*.

Additional processes, such as ecological adaptation and trait shifts, may have further promoted diversification in subclade B1. These could have been influenced by historical introgression, potentially through adaptive introgression of alleles associated with ecological adaptation (e.g., Meier et al., 2017; Svardal et al., 2020) or reproductive isolation (Karimi et al., 2020; Kozak et al., 2021; Stankowski & Streisfeld, 2015). However, in *Picris*, none of the introgression events identified by SNaQ involved the transfer of traits related to life strategy or fruit type (Figure 4), suggesting that adaptive introgression of these traits did not occur.

Alternatively, transgressive segregation following hybridization (Rieseberg et al., 1999; Rosenthal et al., 2002; Yakimowski & Rieseberg, 2014) may have generated novel adaptations or reproductive barriers, ultimately promoting diversification. This process involves recombination between genetically distinct but not overly divergent parental genomes, producing novel, heritable geno‐/pheno‐/ecotypes that exceed the range of parental traits. If such introgression coincides with the availability of unoccupied ecological niches, it may lead to rapid adaptive divergence, as supported by theoretical models (Kagawa & Takimoto, 2018). We hypothesize that the emergence of new *Picris* species in subclade B1 was facilitated by the evolution of novel phenotypes through transgressive segregation. The historical introgression events were likely followed by ecological adaptation to diverse habitats, potentially driving shifts in key traits such as homocarpy and iteroparity. These shifts may have contributed to multiple speciation events at the diploid level. The transition to iteroparity and the colonization of colder temperate zones in subclade B1 may have been accompanied by the emergence of ecophysiological and morphological traits resulting from transgressive segregation (cf. Gross et al., 2004; Lexer et al., 2003; Rosenthal et al., 2002). These traits may involve physiological pathways related to cold tolerance and short growing seasons (Rendón‐Anaya et al., 2021). In the case of hybridization between heterocarpic and semelparous species, historical introgression contributed to the diversification of two morphologically similar lineages within *P. rhagadioloides*, which inhabit hot, dry habitats near the Mediterranean Sea (Lack, 1974; Slovák et al., 2018). Although this introgression did not result in shifts in life strategy, fruit type, or habitat preference (Figure 4), *P. rhagadioloides* is the most widespread Mediterranean endemic, occupying a broad range of dry, often ruderal habitats across the eastern Mediterranean (Lack, 1974; Sell, 1976). It has also been reported as an alien species in certain regions (Galasso et al., 2024), indicating its successful establishment beyond its native range. It is plausible that introgression triggered transgressive trait segregation in this case as well, enabling both intraspecific lineages of *P. rhagadioloides* to become successful and competitive colonizers of dry Mediterranean environments (cf. Souissi et al., 2016).

Finally, our molecular analyses indicate that the taxonomy of Mediterranean members of the genus remains not fully resolved and requires revision. Specifically, our data suggest that several taxa affected by historical introgression have undergone further diversification, and some of these intraspecific lineages may warrant recognition as distinct subspecies or even species. This is particularly evident for *P. cyprica*, which exhibits high genetic variability, as well as for *P. rhagadioloides* or within taxa from the Near East lineage. The greatest challenge, however, will be resolving the complex taxonomy of Asian lineages within the *P. hieracioides* group and their Australian counterparts. Such revisions will require further investigation based on extensive population‐level sampling and an integrated approach combining genomic, morphological, cytological, chorological, and ecological data.

## CONCLUSIONS

Hybridization is widely recognized as a powerful evolutionary force that can drive plant diversification and speciation, with numerous documented cases highlighting its potential to facilitate rapid and adaptive evolutionary radiations. Despite recent advances in genomics and the development of novel analytical approaches, the role of hybridization and introgression in shaping plant diversification remains insufficiently understood, particularly in biodiversity hotspots such as the Mediterranean Basin. In this study, we demonstrate that the diversification of diploid members of the genus *Picris*, centered in the Mediterranean, has been shaped by at least two waves of historical introgression. Specifically, we identified introgression from a relictual Turkish endemic into the MRCAs of two distinct clades. In the case of subclade B1, this event was followed by the emergence of multiple taxa, some of which successfully colonized habitats across three continents. We hypothesize that this historical introgression may have activated genes associated with shifts in life strategy and fruit morphology through transgressive segregation, influencing physiological traits that enhanced adaptation to cold and humid environments. These changes likely promoted both ecological and geographical speciation of *Picris* within and beyond the Mediterranean Basin. Our findings underscore the importance of future research aimed at elucidating the dynamics of transgressive segregation following historical hybridization. Such efforts should integrate whole‐genome data with environmental and ecological information to better understand the mechanisms driving diversification in complex evolutionary landscapes.

## EXPERIMENTAL PROCEDURES

### Sampling design

Our sampling strategy focused on members of Clade B, which are presumed to have been affected by hybridization and introgression (see figure S4a,b in Slovák et al., 2018). The *P. hieracioides* group is represented in our study by accessions of *P. hieracioides* subsp. L. *hieracioides*, *P. hieracioides* subsp. *hispidissima* (Bartl.) Slovák and Kučera, *P. hieracioides* subsp. *umbellata* (Schrank) Ces., *P. olympica* Boiss., *P. japonica* Thunb., and *P. nuristanica Bornm*. Material from the Australian taxa of the *P. hieracioides* group was unavailable for this study. We aimed to analyze at least two individuals per *Picris* taxon. However, for species from the northeastern Mediterranean region, suspected to be predominantly involved in hybridization and exhibiting greater within‐species variability, we included multiple samples per taxon to capture the broadest possible range of intraspecific diversity. Additionally, we included outgroup taxa comprising 17 individuals from 12 genera within the family Compositae. In total, our analysis encompassed 89 individuals representing 43 taxa, of which 72 individuals belonged to 26 *Picris* taxa, from Europe, North Africa, and Southwest Asia (Table S1).

### Karyological analysis

Although most *Picris* taxa are known to be diploid with a chromosome number of 2*n* = 2*x* = 10 (Holzapfel, 1994; Lack, 1974; Slovák et al., 2007, 2018), ploidy information was lacking for some focal species in our study (*P. campylocarpa*, *P. cyprica*, and *P. kotschyi*). Therefore, we conducted chromosome counts to verify their ploidy levels. Chromosome numbers were determined from mitotically active root tips of seedlings and were counted from 1 to 3 seedlings for each species and at least from three mitotic figures per individual. Cypselas from selected populations of *P. campylocarpa*, *P. cyprica*, and *P. kotschyi* (Table S2) were germinated on moist filter paper in Petri dishes. Young roots (~2 mm in length) were collected and pre‐treated in a saturated aqueous solution of *p*‐dichlorobenzene at room temperature for approximately 3 h. The roots were then fixed in a freshly prepared mixture of 96% ethanol and glacial acetic acid (3:1, v/v) and stored at 4°C until further processing. Prior to chromosome preparation, the material was macerated in a 1:1 mixture of ethanol and hydrochloric acid for 30 sec, then transferred to a microscope slide. Non‐meristematic tissues were removed, and the meristem was stained with a drop of lacto‐propionic orcein, covered with a coverslip, and squashed. Preparations were examined using an Olympus BX 51 microscope equipped with a DP‐71 Olympus digital camera and cellSense imaging software v.2.3 (Olympus Corp., Tokyo, Japan).

Chromosome counts confirmed that *P. campylocarpa*, *P. cyprica*, and *P. kotschyi* are diploid, with a chromosome number of 2*n* = 2*x* = 10 (Figure S7).

### Hyb‐Seq data generation

Genomic DNA was extracted from silica gel‐dried leaf tissue using the CTAB protocol (Doyle & Doyle, 1987), with modifications as described by Schönswetter et al. (2002), or alternatively using the DNeasy Plant Mini Kit (Qiagen, Hilden, Germany). DNA extracts with low purity were further purified using the NucleoSpin gDNA Clean‐up Kit (Macherey‐Nagel, Dueren, Germany), following the manufacturer's instructions.

Genomic library preparation for Hyb‐Seq data generation and bait hybridization followed the protocol outlined by Gizaw et al. (2022). The targeted nuclear loci correspond to the Compositae conserved orthologous set (Compositae1061; Mandel et al., 2014), which has previously been validated for *Picris* (Jones et al., 2019). Sequencing was performed on an Illumina NovaSeq 6000 SP platform (San Diego, CA, USA) at the Institute of Applied Biotechnologies (IAB), Olomouc, Czech Republic.

### Data processing and analysis of targeted nuclear loci and off‐target plastid *data*

To analyze targeted nuclear DNA (nDNA) loci, we addressed paralogy using ParalogWizard (Ufimov et al., 2022). To enhance the recovery of both orthologs and paralogs, the Compositae1061 probe set was bioinformatically modified to include sequence representatives from *Picris* prior to segregating sequences into orthologous alignments. These alignments were then processed using the HybPhyloMaker workflow (Fér & Schmickl, 2018) to reconstruct species trees, applying a threshold of ≤50% missing data per accession and ≥75% accession presence per locus. For concatenated analyses, data matrices were generated using AMAS v.1.0 (Borowiec, 2016), and substitution models for each partition (orthologous alignments) were selected based on Akaike Information Criterion (AIC) values computed with ModelTest‐NG v.0.1.6 (Darriba et al., 2020; Flouri et al., 2015).

We generated the following datasets:

*Dataset A*: 89 individuals and 981 concatenated nuclear loci, including 72 *Picris* taxa and 17 outgroup accessions. Used for phylogenetic tree inference.
*Dataset B*: 52 individuals and 999 nuclear loci, comprising 51 *Picris* accessions from Clade B and one individual of *P. sinuata* (Lam.) Lack. from Clade A as the outgroup. It was used for Neighbor‐Net analysis, PhyParts analysis, and coalescent‐based phylogeny for Dsuite.
*Dataset C*: 52 individuals and 1621 nuclear loci, the same taxon composition as Dataset B. Used for variant calling and Dsuite and SNAPP analyses. The number of loci differs from Dataset B because instead of applying filters for ≤50% missing data per accession and ≥75% accession presence per locus, in this case unfiltered loci are used for SNP calling and filtering is subsequently applied to each variant site.
*Dataset D*: 18 individuals and 964 nuclear loci, including 17 *Picris* accessions from Clade B (sensu Slovák et al., 2018) and one individual of *P. sinuata* (Clade A) as the outgroup. *Picris japonica* was grouped with its closest relative, *P. nuristanica*, and *P. rhagadioloide*s was split into two genetic lineages (I and II). It was used for phylogenetic network analysis in SNaQ (Solís‐Lemus et al., 2017).

### cpDNA data

For plastid DNA (cpDNA) analysis, the complete chloroplast genome of *Lactuca tatarica* (GenBank accession number MT845217) was retrieved, and one inverted repeat region was removed. The sequence was split into 119 coding and non‐coding regions. Data processing followed the HybPhyloMaker pipeline. Raw reads were trimmed using Trimmomatic v.0.33 (Bolger et al., 2014), and duplicates were removed with FastUniq v.1.1 (Xu et al., 2012). Reads were mapped to the *L. tatarica* pseudoreference using BWA v.0.7.15 (Li & Durbin, 2009), and consensus variant calling was performed with kindel v.0.1.4 (Constantinides & Robertson, 2017) using a 0.51 threshold. Sequence identity and filtering parameters matched those used for nuclear loci. As with nDNA, coding and non‐coding regions were concatenated using AMAS v.1.0, and the best substitution models for each partition were selected with ModelTest‐NG v.0.1.6.

*Dataset E*: 88 individuals and 119 concatenated plastid loci, including 72 *Picris* taxa and 16 outgroup accessions. Used for plastome phylogeny construction.

### Phylogenomic analyses and species tree inference

Gene trees for each nDNA orthologous alignment were constructed using RAxML v.8.4.2 (Stamatakis, 2014) under the GTR substitution model with gamma‐distributed rate variation among sites (GTRGAMMA) and 500 bootstrap pseudoreplicates.

Coalescent‐based nDNA phylogenies were inferred from datasets A and B using ASTRAL‐III v.5.7.4 (Zhang et al., 2018), treating all accessions as terminal taxa. Additionally, maximum likelihood phylogenies for both nDNA (dataset A) and cpDNA (dataset E) concatenated datasets were generated using RAxML‐NG v.8 (Kozlov et al., 2019), applying the best‐fit model for each partition and performing bootstrapping with up to 1000 pseudoreplicates. Bootstrapping converged after 50 replicates for dataset A and 250 replicates for dataset E, using the transfer bootstrap expectation method (Lemoine et al., 2018).

To assess gene tree concordance and conflict, PhyParts (Smith et al., 2015) was used to count consistent and conflicting bipartitions for key lineages in the coalescent‐based phylogeny inferred from dataset B. This analysis aimed to identify nodes potentially affected by introgression. Trees were rooted with *P. sinuata* using Newick Utilities (Junier & Zdobnov, 2010), and branches with bootstrap support below 50% were collapsed. Resulting pie charts were mapped onto the ASTRAL species tree using the script ‘phypartspiecharts.py’ (https://github.com/mossmatters/MJPythonNotebooks).

Phylogenetic relationships within Clade B were also reconstructed using a Bayesian framework implemented in SNAPP v.1.6.1 (Leaché et al., 2014) within BEAST v.2.7 (Bouckaert et al., 2019). Variant calling was performed on loci from dataset C, applying a 20% maximum missing data threshold per site. After retaining one SNP per locus, 1425 SNPs remained. Model parameters and priors followed the recommendations of Leaché and Bouckaert (2018). Three independent Markov chain Monte Carlo (MCMC) chains were run for 100 million generations, with 10% burn‐in and sampling every 1000 generations. Chains were combined using LogCombiner v.2.7.7 available in BEAST, and convergence was assessed in Tracer v.1.7.1 (Rambaut et al., 2018), with effective sample sizes exceeding 200 for most parameters. A maximum clade credibility tree with common ancestor heights was generated using TreeAnnotator v.2.7.7 (Suchard et al., 2018).

### Divergence time estimation

Divergence times within *Picris* Clade B were estimated using penalized likelihood implemented in treePL v.1.0 (Sanderson, 2002; Smith & O'Meara, 2012), following the guidelines of Maurin (2020). The concatenated nuclear phylogeny generated from dataset A was pruned using the package ape v.5.7 (Paradis & Schliep, 2019) in R v.3.5 (R Core Team, 2022), retaining only accessions from Clade B and *P. sinuata* as the outgroup. Three secondary calibration points were applied, based on the 95% confidence interval of the highest posterior density values estimated by Slovák et al. (2018): stem age of Clade B = 6.9–9.3 Mya, crown age of Clade B = 4.1–6.5 Mya, and crown age of the *P. hieracioides* group = 0.9–2.6 Mya. The original divergence time estimates were based on a combination of primary and secondary calibration points, with fossil calibration derived from Tremetsberger et al. (2013). The pruned nuclear phylogeny and a TreePL wrapper script (https://github.com/tongjial/treepl_wrapper) were used for parameter optimization and cross‐validation. To estimate confidence intervals for node ages, 200 bootstrapped phylogenies, maintaining the same topology as the pruned tree, were dated using the same calibration points and optimized parameters. The resulting dated trees were summarized using TreeAnnotator v.2.6.4.

### Reticulation analyses

To evaluate reticulations, several analytical approaches were applied, focusing exclusively on datasets containing members of Clade B, with *P. sinuata* serving as the outgroup.

A distance‐based network was constructed from dataset B using Neighbor‐Net (Bryant & Moulton, 2004), implemented in SplitsTree v.4.16.2 (Huson et al., 2008). Accessions with high levels of missing data (potentially introducing artificial reticulations) were excluded from visualization (*P_galilaea*_IZ_11_8, *P_nuristanica*_NUR8, and *P_strigosa*_P1_2).

Historical introgression was assessed using the *f*‐branch statistic, implemented in Dsuite v.0.5 (Malinsky et al., 2021), based on SNP data. A coalescent‐based topology for dataset B was generated using ASTRAL, as described above. Loci from dataset C were concatenated, and variant sites were called using snp‐sites v.2.3.3 (Page et al., 2016), with *P. sinuata* as the reference. Because the resulting VCF file did not properly handle missing data (alignment gaps), it was modified using custom commands (as implemented in the HybPhyloMaker8i script) to correctly introduce missing genotypes (./.). The modified VCF file was filtered using bcftools v.1.17 (Li, 2011) to retain only biallelic SNPs with <20% missing data. A single SNP per 100 base pairs was retained using vcftools v.1.6 (Danecek et al., 2011). The final filtered and thinned VCF file contained 2390 SNPs and was used as input for Dsuite alongside the coalescent‐based topology from dataset B. The *f*‐branch (*f*_b_) statistic (Malinsky et al., 2018) was computed to summarize f4‐ratios across the tree topology, using a significance threshold of *P* value ≤0.05. To control for family‐wise error, *P* values were adjusted using the False Discovery Rate (FDR) method (Jafari & Ansari‐Pour, 2019).

To visualize relatedness among samples, a pairwise matrix of simple‐matching similarity coefficients was calculated using a custom R script (‘SNPheatmap.R’ from HybPhyloMaker) and plotted as a heatmap using the gplots package in R (Warnes et al., 2024). The binary matrix was derived from the VCF file used in the Dsuite analysis. Reticulation events were identified using SNaQ, which estimates phylogenetic networks under the multispecies coalescent model within a pseudolikelihood framework, accounting for ILS and enabling the representation of reticulate histories (Solís‐Lemus & Ané, 2016). To reduce computational demands, dataset D was used, comprising one individual per taxon from Clade B and *P. sinuata* as the outgroup. Five different resampling sets were created to account for within‐species variability, prioritizing individuals with the lowest proportion of missing data. Gene trees from the ASTRAL phylogeny were used as scaffolds for SNaQ network construction. To minimize noise, gene trees were filtered using two criteria: (1) the maximum distance among in‐group taxa must not exceed 1.5 times the maximum distance between ingroup taxa and *P. sinuata*, and (2) the minimum distance between ingroup taxa and *P. sinuata* must be ≥0.0001. Branch lengths of filtered trees were normalized using the mean distance between *P. sinuata* and ingroup taxa. Depending on the resampling set, 964–980 of the 1033–1075 gene trees met these criteria and were used to build SNaQ networks. Two independent SNaQ runs were performed for each resampling, each with 10 replicates. For each run, five analyses were conducted allowing for zero (hmax = 0) to five (hmax = 5) introgression events. All candidate networks obtained by rotating the reticulation cycle in the highest‐pseudolikelihood network were also considered (https://solislemuslab.github.io/snaq‐tutorial/lecture‐notes/lecture4.html). Candidate networks were evaluated separately for each number of reticulations. Two types of networks were excluded a priori: (1) those involving the outgroup in any reticulation, and (2) those with pseudolikelihood values ≥30 log units lower than the best network from the same resampling and reticulation level (hereafter referred to as the reference network). The remaining candidate networks, rooted with *P. sinuata*, were assessed based on pseudolikelihood support: (1) strong when ≤10 log units lower than the reference network, (2) moderate when 10–20 log units lower, and (3) poor when 20–30 log units lower. The prevailing network topology was selected using a majority rule, favoring the network with the highest number of reticulations and strong or moderate support across the five resamplings. Networks with the same number of reticulations and strong or moderate support from at least two resamplings were also considered.

All reticulation events from the selected networks were tested to determine whether they resulted from introgression or ILS (Owens et al., 2023). For each putative introgression, species were categorized into four groups (Figure S8). For the four groups, only three possible tree topologies exist: a major and a minor topology (related to the reticulation node), and a third, contradictory topology not represented in the network. TWISST (Martin & Van Belleghem, 2017) was used to calculate the frequency of each topology across gene trees. The TWISST script was customized to account for gene trees of unequal size. A majority rule was applied to assign support from each gene tree; if multiple topologies were supported, the support was split equally. Counts of gene trees supporting the minor and contradictory topologies were compared using a chi‐square test under the null hypothesis that ILS would result in equal frequencies.

### Network calibration

To compute terminal branch lengths and ultrametrize the selected networks, a calibration procedure was performed using the calibrateFromPairwiseDistances! function (Solís‐Lemus et al., 2017) from the Julia package PhyloNetworks v0.16.4 (Bastide et al., 2018; Karimi et al., 2020). This function was customized (calibrateFromPairwiseDistances‐ext.jl) to minimize not only the differences between pairwise distances in the network and the mean pairwise distances from gene trees, but also changes in the lengths of internal branches. Because branch lengths estimated by SNaQ do not correlate well with calendar time (Solís‐Lemus et al., 2017), a power transformation was applied to branch lengths using a given or estimated exponent. This approach helps prevent signal loss from SNaQ, which often results in extremely short or even zero‐length internal branches. Expanding the optimization objective function also enabled the search for global rather than local optima.

For calibration, a matrix of mean pairwise distances between taxa was prepared from the same set of normalized gene trees used for SNaQ network inference. While Karimi et al. (2020) proposed a similar normalization procedure using the median, we used the mean. These mean pairwise distances were further adjusted to match the internal branch lengths of the SNaQ networks after applying the power function with an initial exponent (*p*_init_). The exponent was automatically optimized within a range of [*p*_init_/10, *p*_init_ × 10]. To compensate for the inherently shorter internal branches, the cost of internal branch length changes was weighed by the ratio of the mean value of the pairwise distance matrix to the mean internal branch length of the network.

### Reconstruction of discrete trait evolution

The evolutionary history of one extrinsic trait (environmental predictability) and two intrinsic traits (longevity and fruit morphology) was reconstructed for 18 *Picris* taxa using the selected, calibrated SNaQ networks. For intrinsic traits, taxa were categorized according to their trait states as follows: with respect to fruit morphology, species were classified as either homocarpic or heterocarpic, whereas for life strategy, taxa were assigned to semelparous (annual) or iteroparous (perennial) categories. In terms of the extrinsic trait, the studied taxa were assigned to habitats characterized by either predictable or unpredictable environmental conditions. This classification was primarily based on annual precipitation patterns, following Imbert (2002), with modifications to account for the specific characteristics and biotope diversity of the study area (cf. Slovák et al., 2018). Predictable habitats included biomes of the boreal and temperate regions of Europe and Asia, as well as alpine environments in the Mediterranean region (e.g., *P. olympica*), whereas Mediterranean lowland biotopes, semi‐deserts, and deserts were classified as unpredictable habitats. Climatic zones were delineated according to the Bioclimates of the World framework within the Worldwide Bioclimatic Classification System (1996–2016; Rivas‐Martinez & Rivas‐Saenz, 2016).

Analyses were conducted under the Equal Rates Substitution Model (ERSM) implemented in PhyloNetworks (Bastide et al., 2018; Karimi et al., 2020). Character states and their codings are summarized in Table S3.

## AUTHOR CONTRIBUTIONS

The conception and design of the study were carried out by MS and RES. Data collection was conducted by JK, AAD, İSY, and ZUA. Karyological analysis was performed by ML. Data analysis, plotting, and the description of the methods were completed by JMG, TF, IR, and PV. The first draft of the manuscript was written by MS and RES, with all authors providing feedback on prior versions. All authors reviewed and approved the final manuscript.

## CONFLICT OF INTEREST

The authors declare no conflicts of interest.

## Supporting information


**Figure S1.** Distribution maps of selected *Picris* species analyzed in this study, originating from the Mediterranean region.
**Figure S2.** Maximum likelihood tree generated using RAxML‐NG, based on 981 concatenated nuclear loci and 89 individuals from the genus *Picris* and the outgroup.
**Figure S3.** Bayesian coalescent‐based species tree generated using SNAPP, based on 1425 single nucleotide polymorphisms and 52 *Picris* accessions.
**Figure S4.** PhyPart assessment of phylogenomic signal based on 999 nuclear loci and 52 individuals within *Picris* Clade B, with *P. sinuata* as the outgroup.
**Figure S5.** Divergence time estimation for *Picris* Clade B performed on the pruned concatenated nuclear phylogeny using penalized likelihood in treePL. *Picris sinuata* was used as the outgroup.
**Figure S6.** Distance‐based network constructed using the Neighbor‐Net algorithm, based on 52 *Picris* individuals and 999 nuclear loci.
**Figure S7.** Mitotic metaphases of selected *Picris* species from Türkiye. (a) *P. cyprica*, 2*n* = 2*x* = 10 (locality TR6). (b) *P. campylocarpa*, 2*n* = 2*x* = 10 (locality TR10). (c) *P. kotschyi*, 2*n* = 2*x* = 10 (locality TR15).
**Figure S8.** Delimitation of taxon groups used in TWISST analysis.
**Table S1.** List of populations of the studied *Picris* and outgroup taxa, including population codes, collection data, chromosome numbers, and GenBank accession numbers.
**Table S2.** List of sampled populations used for karyological analyses.
**Table S3.** List of characters used for ancestral state reconstructions.

## Data Availability

Data underlying this study have been made freely available through the Open Science Framework (OSF) Repository at: https://doi.org/10.17605/OSF.IO/DJ93K. Sequencing data have been deposited in the NCBI Sequence Read Archive under the BioProject accession number PRJNA1193563, with individual accession numbers SRR31603548–SRR31603626.
