# Supplementary material for: Historical introgression as a driver of diversification of diploid Picris (Compositae) in the Mediterranean Basin
Source: Plant J. 2025 Nov 11;124(3):e70565. doi: 10.1111/tpj.70565 (PMC12604549; doi:10.1111/tpj.70565)
Supplement: Supplementary file 1 — Figure S1. Distribution maps of selected Picris species analyzed in this study, originating from the Mediterranean region. Figure S2. Maximum likelihood tree generated using RAxML‐NG, based on 981 concatenated nuclear loci and 89 individuals from the genus Picris and the outgroup. Figure S3. Bayesian coalescent‐based species tree generated using SNAPP, based on 1425 single nucleotide polymorphisms and 52 Picris accessions. Figure S4. PhyPart assessment of phylogenomic signal based on 999 nuclear loci and 52 individuals within Picris Clade B, with P. sinuata as the outgroup. Figure S5. Divergence time estimation for Picris Clade B performed on the pruned concatenated nuclear phylogeny using penalized likelihood in treePL. Picris sinuata was used as the outgroup. Figure S6. Distance‐based network constructed using the Neighbor‐Net algorithm, based on 52 Picris individuals and 999 nuclear loci. Figure S7. Mitotic metaphases of selected Picris species from Türkiye. (a) P. cyprica, 2n = 2x = 10 (locality TR6). (b) P. campylocarpa, 2n = 2x = 10 (locality TR10). (c) P. kotschyi, 2n = 2x = 10 (locality TR15). Figure S8. Delimitation of taxon groups used in TWISST analysis. Table S1. List of populations of the studied Picris and outgroup taxa, including population codes, collection data, chromosome numbers, and GenBank accession numbers. Table S2. List of sampled populations used for karyological analyses. Table S3. List of characters used for ancestral state reconstructions. [file TPJ-124-0-s001.docx]

**Supplemental Information for**

**Historical introgression as a driver of diversification of diploid *Picris* (Compositae) in the Mediterranean Basin**

**Juan Manuel Gorospe^1,2,†^, Tomáš Fér^1,†^, Ivan Rurik^3,†^, Peter Vďačný^3^, Jaromír Kučera^4^,** [**Ali A. Dönmez**](https://onlinelibrary.wiley.com/authored-by/D%C3%B6nmez/Ali+A.)**^5^, İbrahim** [**Sırrı Yüzbaşıoğlu**](https://onlinelibrary.wiley.com/authored-by/Y%C3%BCzba%C5%9F%C4%B1o%C4%9Flu/S%C4%B1rr%C4%B1)**^6^, Zübeyde Uğurlu Aydın^5^, Magdalena Lučanová^2,7^, Roswitha Elisabeth Schmickl^1,2,*^ and Marek Slovák^1,4,*^**

*^1^Department of Botany, Faculty of Science, Charles University, 128 01 Prague, Czechia,*

*^2^Department of Evolutionary Plant Biology, Institute of Botany of the Czech Academy of Sciences, 252 43 Průhonice, Czechia,*

*^3^Department of Zoology, Comenius University Bratislava, 842 15 Bratislava, Slovakia,*

*^4^Institute of Botany, Plant Science and Biodiversity Centre, Slovak Academy of Sciences, 845 23 Bratislava, Slovakia,*

*^5^Molecular Plant Systematic Laboratory (MOBIS), Department of Biology, Faculty of Science, Hacettepe University, Ankara, Turkey,*

*^6^Department of Botany, Faculty of Science, İstanbul University, İstanbul, Turkey, and*

*^7^Department of Botany, Faculty of Science, University of South Bohemia, 370 05 České Budějovice, Czechia*

*For correspondence (e-mails roswitha.schmickl@natur.cuni.cz and marek.slovak@savba.sk).

†These authors contributed equally to this work.

**
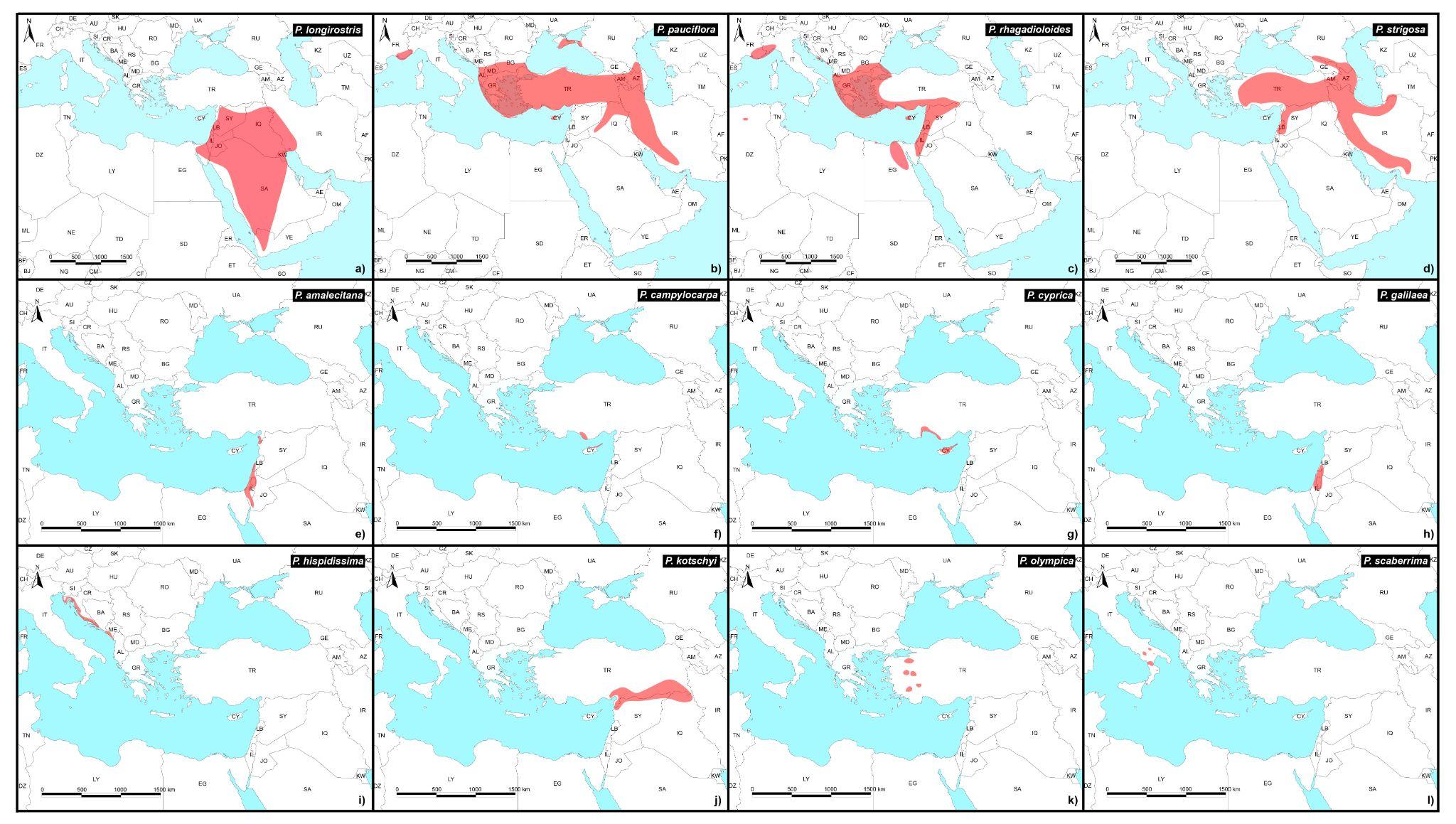
**

**Figure S1.** Distribution maps of selected *Picris* species analyzed in this study, originating from the Mediterranean region. The broader Eurasian distribution of *Picris hieracioides* subsp. *hieracioides* and *Picris hieracioides* subsp. *umbellata*, as well as the distribution of analyzed Asian taxa within the *Picris hieracioides* group, are not depicted. Similarly, distribution data for *Picris rhagadioloides* occurring in Morocco and the Iberian Peninsula are excluded from this visualization.


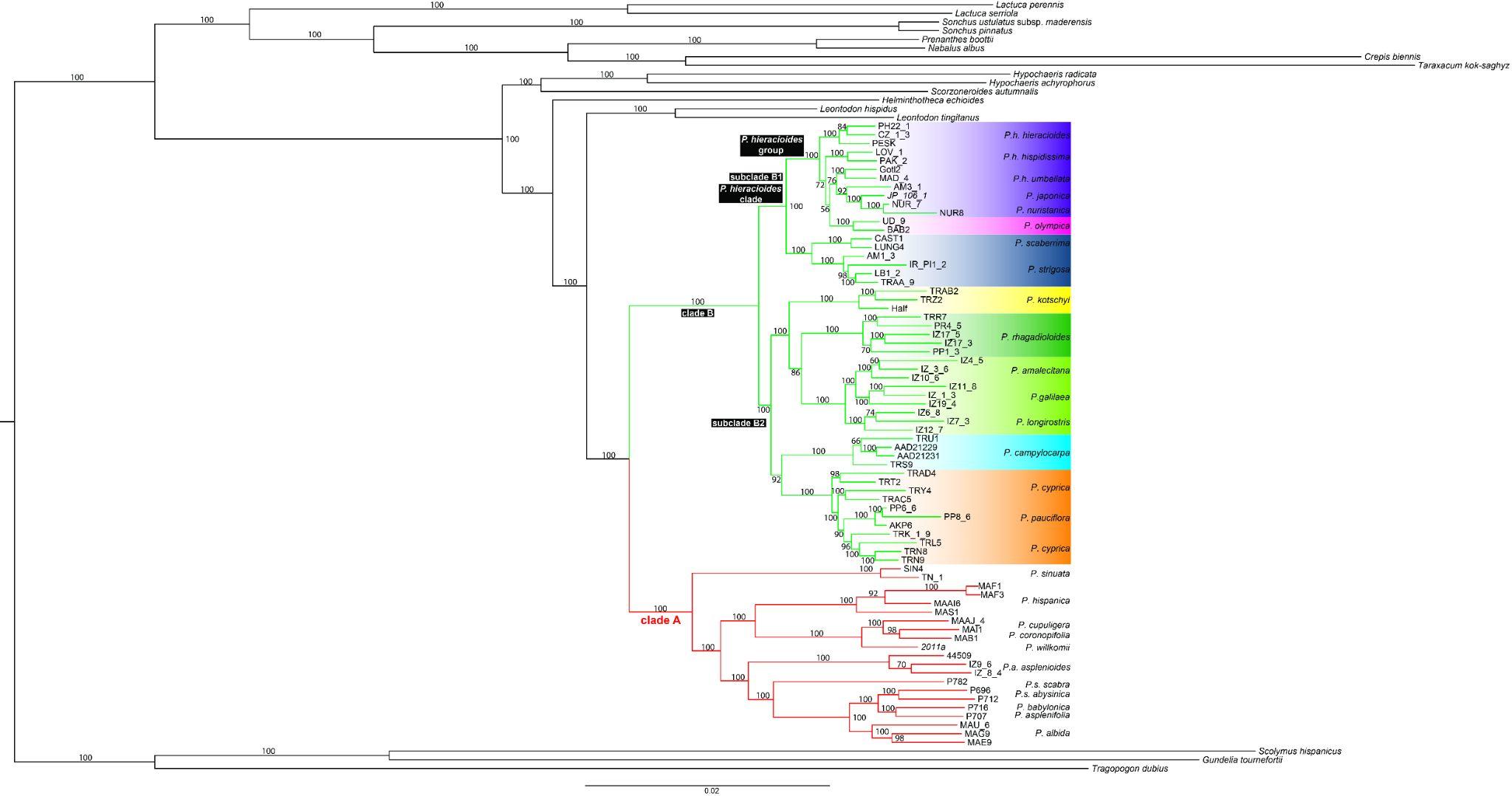


**Figure S2.** Maximum likelihood tree generated using RAxML-NG, based on 981 concatenated nuclear loci and 89 individuals from the genus *Picris* and the outgroup. Bootstrap support values are shown above branches.


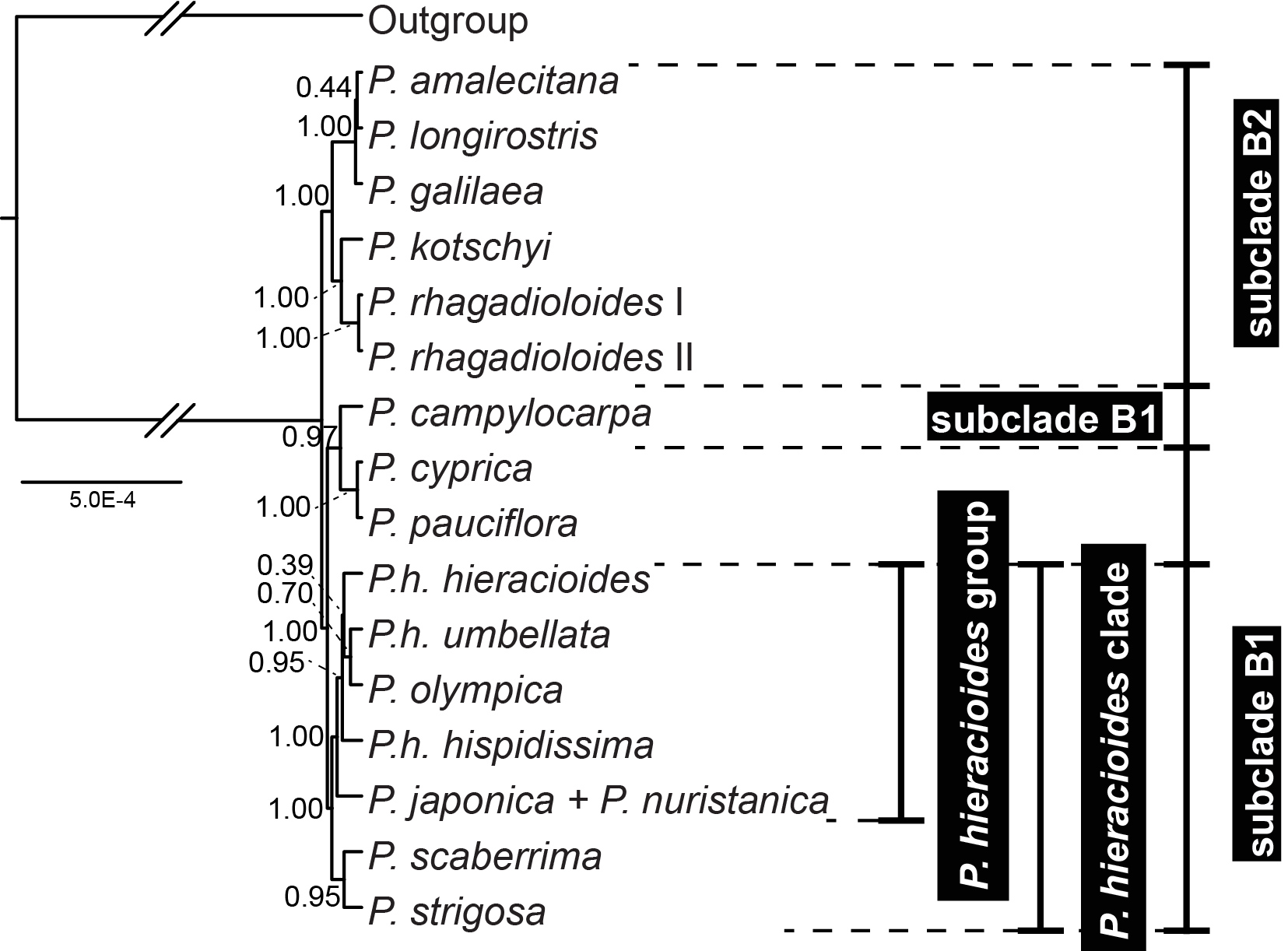


**Figure S3.** Bayesian coalescent-based species tree generated using SNAPP, based on 1425 single nucleotide polymorphisms and 52 *Picris* accessions. *Picris japonica* is represented by a single individual and was grouped with the accessions of the closely related *P. nuristanica*. Posterior probability values are shown above branches.


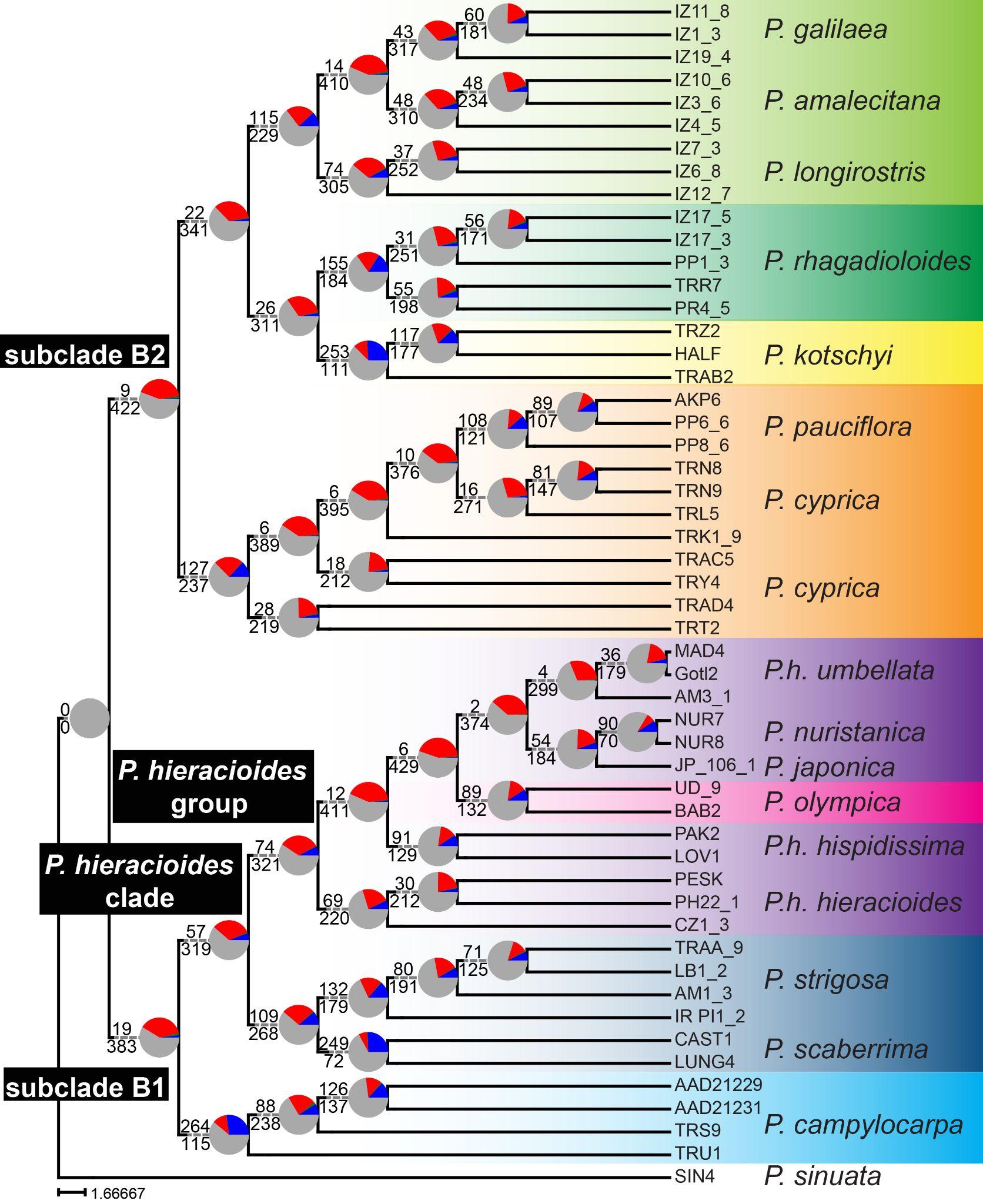


**Figure S4.** PhyParts assessment of phylogenomic signal based on 999 nuclear loci and 52 individuals within *Picris* Clade B, with *P. sinuata* as the outgroup. Pie charts illustrate the degree of topological concordance and conflict at each node, showing the proportion of gene trees in concordance (blue), in conflict (red), and uninformative (grey). Numbers above and below the branches indicate the counts of concordant and conflicting gene trees, respectively.


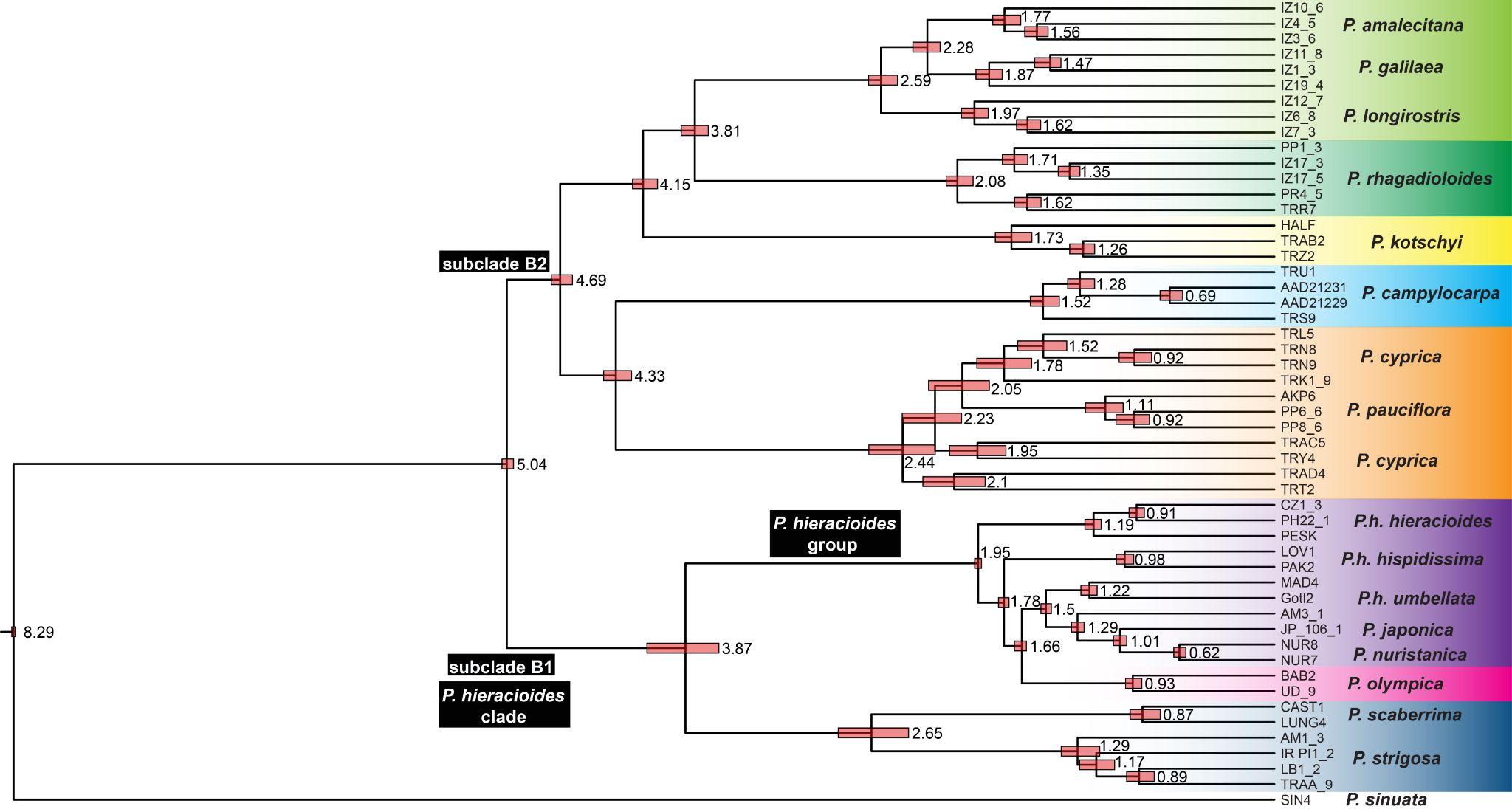


**Figure S5.** Divergence time estimation for *Picris* Clade B performed on the pruned concatenated nuclear phylogeny using penalized likelihood in treePL. *Picris sinuata* was used as the outgroup.


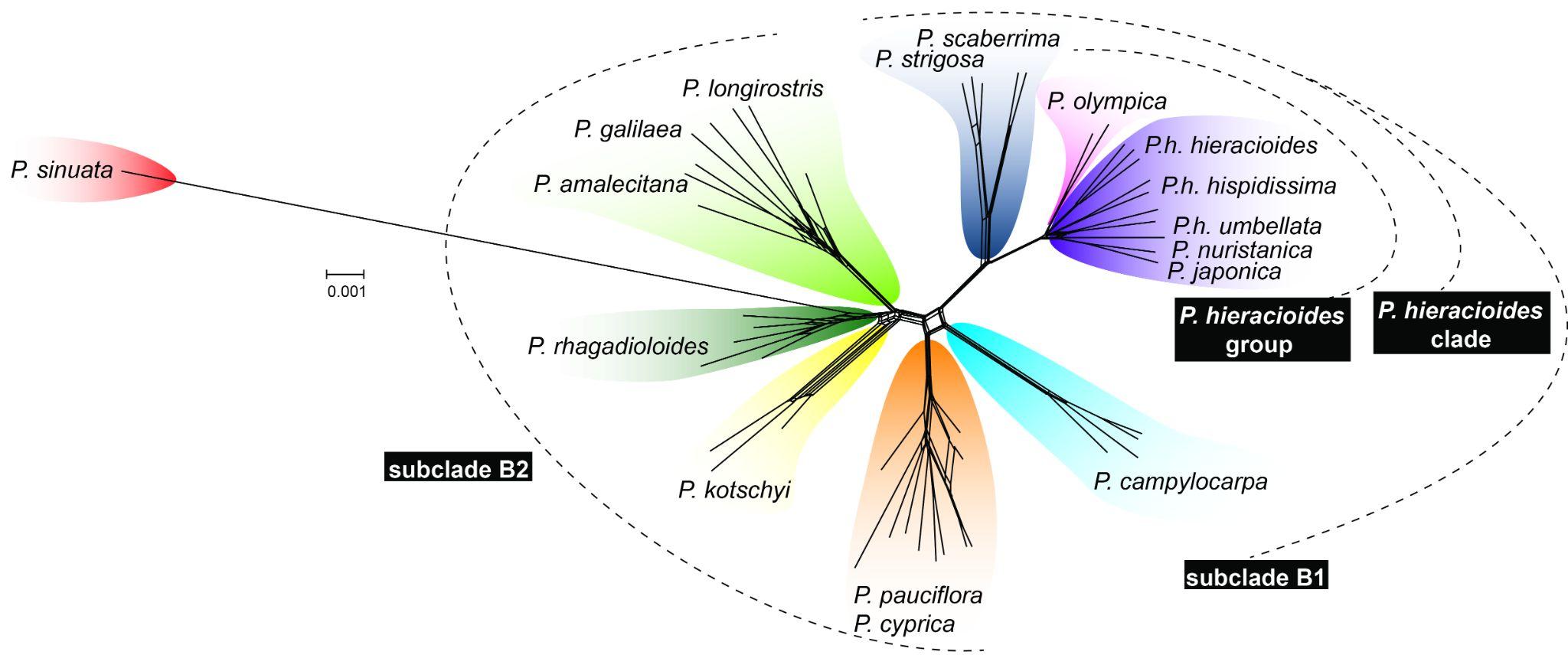


**Figure S6.** Distance-based network constructed using the Neighbor-Net algorithm, based on 52 *Picris* individuals and 999 nuclear loci.

**
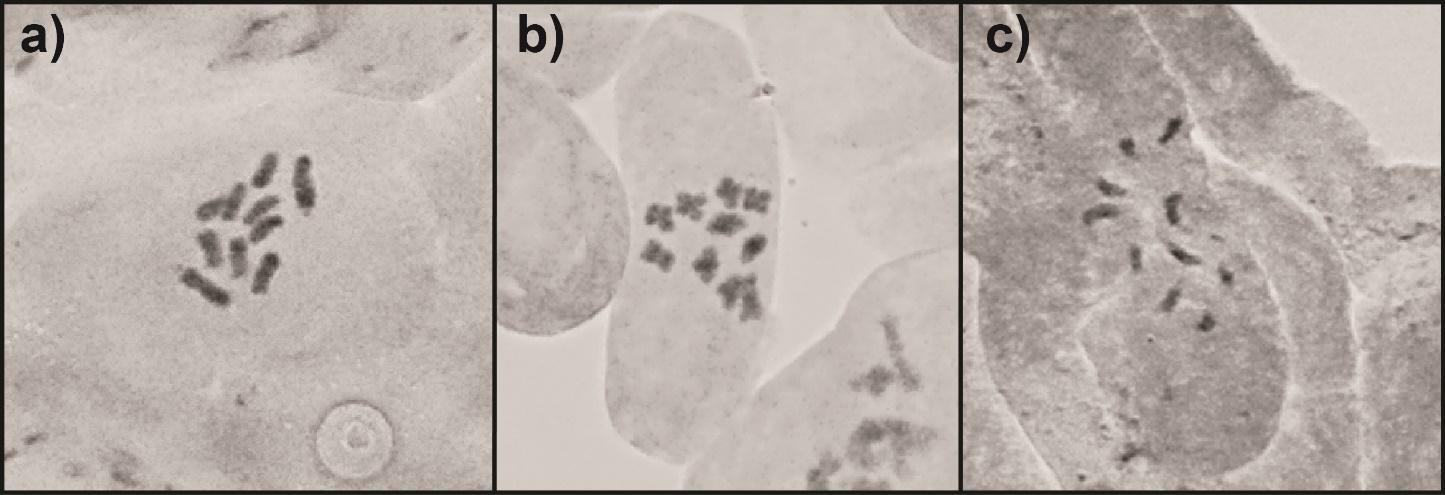
**

**Figure S7.** Mitotic metaphases of selected *Picris* species from Türkiye. (a) *P. cyprica*, 2n = 2x = 10 (locality TR6). (b) *P. campylocarpa*, 2n = 2x = 10 (locality TR10). (c) *P. kotschyi*, 2n = 2x = 10 (locality TR15). For locality codes and details, see Supplementary Table S2.


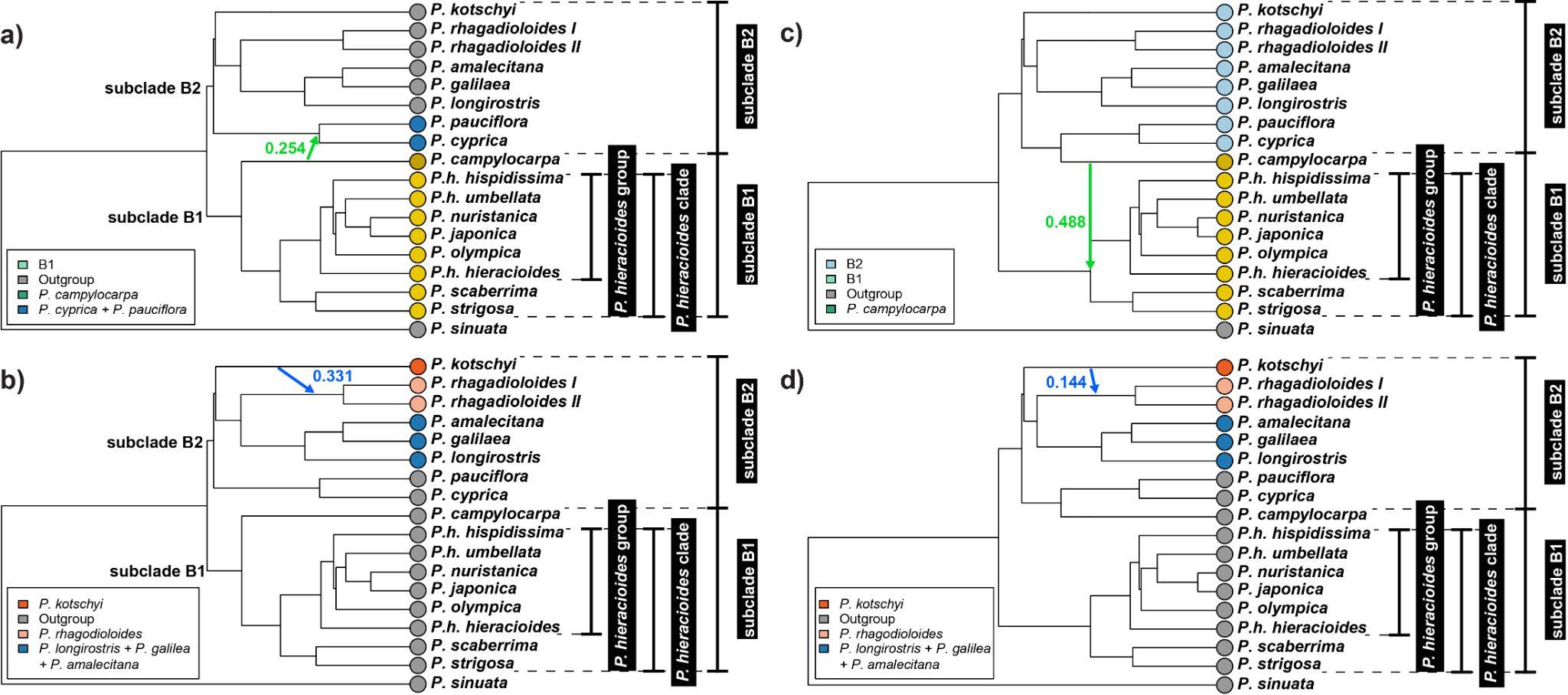


**Figure S8.** Delimitation of taxon groups used in TWISST analysis. Four groups of taxa (indicated by different colors) were defined to test gene tree support for two alternative topologies associated with two reticulation events.

**Table S1.** List of populations of the studied *Picris* and outgroup taxa, including population codes, collection data, chromosome numbers, and GenBank accession numbers. Sequences of taxa marked with an asterisk (*) were retrieved from GenBank (NCBI – study by Mandel et al., 2019); those marked with two asterisks (**) were retrieved from GenBank (NCBI – study by Jones et al., 2019); and the accession marked with a hashtag (#) were cultivated from seeds obtained from the Millennium Seed Bank, Royal Botanic Gardens, Kew (Great Britain).Abbreviations: N.A., data not available; ADH, anthropogenically disturbed habitats. If detailed coordinates were missing from herbarium specimens, they were georeferenced and are shown in square brackets. Chromosome number data were compiled from the following sources: Astuti et al. (2015), Berjano et al. (2014), Galland (1988), Hong and Zhang (1999), Holzapfel (1994), Huang and Ge (1988), Humphries (1978), Kučera and Slovák (2012), Kučera et al. (2016), Lack (1974, 1977), Luque and Lifante (1991), Malallah and Brown (1999), Murray and Lange (1999), Nazarova (1984), Oberprieler and Vogt (1993), Peng and Hsu (1978), Podlech (1986), Probatova (2005), Slovák et al. (2007, 2009a, 2009b, 2012, 2014), Smalla (2000), Stepanov (1994), and Talavera (1979). Ecological data for individual taxa were obtained from the authors’ personal field observations and/or the following sources: Lack (1974, 1979), Holzapfel and Lack (1993), Smalla (2000), and Slovák et al. (2009a, 2009b, 2012, 2014).

| **Taxon** | **Locality** | **Chromosome number** | **Biotope** | **GenBank accession entries** |
| --- | --- | --- | --- | --- |
| *P. albida* Ball |  | unknown |  |  |
| MAU6 | MA, Souss-Massa Draa region, Jbel Bani saddleback, above Anagam village, 862 m, 30°07′11′′N, 5°37′52′′W, col. M. Slovák, J. Kučera, A. Guttová and K. Olšavská, 6.5.2013 (SAV) |  | Rocky deserts and semideserts, dry mountain rocky slopes |  |
| MAG9 | MA, Souss-Massa Draa region, Tafraout village, 2km S of thevillage, 1037 m, 29°42′23′′N,8°58′03′′W, col. M. Slovák, J. Kučera, A. Guttová and K. Olšavská, 3.5.2013 (SAV) |  | Rocky deserts and semideserts, dry mountain rocky slopes |  |
| MAE9 | MA, Souss-Massa Draa region, near Ida Ougnidif village, 1210 m, 29°51'11.900"N, 9°0'13.600"W, col. M. Slovák, J. Kučera, A. Guttová and K. Olšavská, 2.5.2013 (SAV) |  | Rocky deserts and semideserts, dry mountain rocky slopes |  |
| *P. amalecitana* (Boiss.) Eig |  | unknown |  |  |
| IZ10/6 | IL, Center district, near Michmoret village, Alexander river park, 4 m, 32°23′43′′N, 34°52′20′′E, col. M. Slovák and J. Kučera, 3.4.2012 (SAV) |  | Calcareous coastal sandy dunes, ADH |  |
| IZ3/6 | IL, Tel Aviv district, Kurkar National Park, Nes-Zïaona, 44 m, 31°55′59′′N, 34°47′09′′E, col. M. Slovák and J. Kučera, 1.4.2012 (SAV) |  | Calcareous coastal sandy dunes, ADH |  |
| IZ4/5 | IL, Southern district, Karmyya, Carmia Sands Nature reserve, 27 m, 31°35′31′′N, 34°32′29′′E, col. M. Slovák and J. Kučera, 1.4.2012 (SAV) |  | Calcareous coastal sandy dunes, ADH |  |
| *P. asplenioides* L. subsp. *asplenioides* |  | 2*n* = 10 |  |  |
| IZ9/6 | IL, Southern district, near petrol station S of Ramat Khovav village, 362 m, 31°06′18′′N, 34°49′25′′E, col. M. Slovák and J. Kučera, 2.4.2012 (SAV) |  | Sandy dunes in inland desert and semidesert |  |
| Iz8/4 | IL, Southern district, E of Dimona town, 419 m, 31°02′21′′N, 35°09′32′′E, col. M. Slovák and J. Kučera, 2.4.2012 (SAV) |  | Sandy dunes in inland desert and semidesert |  |
| 44509 | EG, between Idku and Rosetta villages, col. D. Podlech 44509, 2.2.1989 (MSB) |  | Sandy dunes in inland desert and semidesert |  |
| P707 | SA, Shafa highland, 30 km SW of Taif, 1830-2050 m, col. A.A. Fayed 1341 (det. as Picris longirostris), 30.1.1988 (MSB) |  | Sandy dunes in inland desert and semidesert |  |
| *P. babylonica* Hand.-Mazz. |  | 2*n* = 10 |  |  |
| P716 | SA, [Aš Šarqijah], Eastern Province: 27 km WSW Al-Qaysumah, 28°14′N, 45°51′E, col. D Hillcoat 306, 26.3.1981 (BM 81273) |  | Lowland sands, calcareous rocky deserts and semideserts |  |
| *P. campylocarpa* Boiss. et Heldr. |  | 2*n* = 10 |  |  |
| AAD21231 + AAD21229 | CY, Girne, near Esentepe village, 172 m, 35°20'43.0''N, 33°40'21.6''E, col. Z. Uğurlu and A Ünlü, 13.5.2022 (SAV) |  | Xerothermous lowland, silicate rocky slopes and grasslands, ADH |  |
| TRS9 | TR, Antalya province, near Taşkesiği village, 168 m, 36°21′57′′N, 32°11′53′′E, col. M. Slovák and J. Kučera, 21.5.2012 (SAV) |  | Xerothermous lowland, silicate rocky slopes and grasslands, ADH |  |
| TRU1 | TR, Antalya province, near Yeşilöz village, 23 m, 36°45′09′′N, 31°37′28′′E, col. M. Slovák and J. Kučera, 21.5.2012 (SAV) |  | Xerothermous lowland, silicate rocky slopes and grasslands, ADH |  |
| *P. coronopifolia* DC. |  | unknown |  |  |
| MAI1 | MA, Guelmin Es Semara region, Lakhsas village, ca. 3km N of the village, 1037 m, 29°24′05′′N, 9°43′46′′W, col. M. Slovák, J. Kučera, A. Guttová and K. Olšavská, 3.5.2013 (SAV) |  | Xerothermous lowland, midaltitudinal calcareous rocky slopes and semideserts |  |
| *P. cupuligera* (Durieu) Walp. |  | 2*n* = 10 |  |  |
| MAAJ4 | MA, Fes Boulemane region, near Oulad Ayyad village, 275 m, 34°05′42′′N, 4°36′17′′W, col. M. Slovák, J. Kučera, A. Guttová and K. Olšavská, 11.5.2013 (SAV) |  | Xerothermous lowland, midaltitudinal rocky slopes and grasslands, ADH |  |
| MAB1 | MA, Rabat Salé Kenitra region, Marchouch village, ca. 1,5km E of the village, 400 m, 33°34′01′′N,6°42′59′′W, col. M. Slovák, J. Kučera, A. Guttová and K. Olšavská, 30.4.2013 (SAV) |  | Xerothermous lowland, midaltitudinal rocky slopes and grasslands, ADH |  |
| *P. cyprica* Lack |  | 2*n* = 10 |  |  |
| TRAD4 | TR, Antalya province, Iotape ruins near Gazipaşa, 26 m, 36°19′11′′N, 32°14′11′′E, col. M. Slovák and J. Kučera, 26.5.2012 (SAV) |  | Xerothermous, coastal calcareous rocky slopes and grasslands, ADH |  |
| TRN8 + TRN9 + TR3 | TR, Antalya province, Kaputaş canyon, near Kalkan town, 5 m, 36°13′46′′N, 29°26′59′′E, col. M. Slovák and J. Kučera, 19.5.2012 (SAV) and col. M. Slovák and J. Kučera, 11.6.2023 (SAV) |  | Xerothermous, coastal calcareous rocky slopes and grasslands, ADH |  |
| TRK1/9 | TR, Antalya province, near Çamyuva village, 78 m, 36°31′48′′N, 30°32′58′′E, col. M. Slovák and J. Kučera, 18.5.2012 (SAV) |  | Xerothermous, coastal calcareous rocky slopes and grasslands, ADH |  |
| TRT2 + TR8 | TR, Antalya province, near Murtiçi village, 524 m, 36°53′43′′N, 31°45′31′′E, col. M. Slovák and J. Kučera, 21.5.2012 (SAV) and col. M. Slovák and J. Kučera, 11.6.2023 (SAV) |  | Xerothermous, coastal calcareous rocky slopes and grasslands, ADH |  |
| TRAC5 | TR, Mersin province, near Tokmar Kalesi castle, 275 m, 36°15′43′′N, 33°47′36′′E, col. M. Slovák and J. Kučera, 25.5.2012 (SAV) |  | Xerothermous, coastal calcareous rocky slopes and grasslands, ADH |  |
| TRY4 | TR, Mersin province, near Aydincin village, 282 m, 36°09′29′′N, 33°24′07′′E, col. M. Slovák and J. Kučera, 22.5.2012 (SAV) |  | Xerothermous, coastal calcareous rocky slopes and grasslands, ADH |  |
| TRL5 | TR, Antalya province, near Yenbey village, 391 m, 36°25′52′′N, 30°25′34′′E, col. M. Slovák and J. Kučera, 18.5.2012 (SAV) |  | Xerothermous, coastal calcareous rocky slopes and grasslands, ADH |  |
| TR6 | TR, Antalya province, Aydinlar, near road to Termessos, 350 m, 37.020378°N, 30.511417°E, col. M. Slovák and J. Kučera, 11.6.2023 (SAV) |  | Xerothermous, coastal calcareous rocky slopes and grasslands, ADH |  |
| *P. galilaea* (Boiss.) Eig |  | unknown |  |  |
| IZ1/3 | IL, Jerusalem district, near Messilat Zion village, 332 m, 31°47′45′′N, 35°00′56′′E, col. M. Slovák and J. Kučera, 29.3.2012 (SAV) |  | Xerothermous, calcareous rocky slopes and grasslands in semidesert mountains |  |
| IZ11/8 | IL, Haifa district, Carmel Mt., near Etsba Cave, 72 m, 32°42′47′′N, 34°58′34′′E, col. M. Slovák and J. Kučera, 3.4.2012 (SAV) |  | Xerothermous, calcareous rocky slopes and grasslands in semidesert mountains |  |
| IZ19/4 | IL, Golan Heights district, Nahal Meitsar nature reserve, 290 m, 32°46′06′′N, 35°41′33′′E, col. M. Slovák and J. Kučera, 8.4.2012 (SAV) |  | Xerothermous, calcareous rocky slopes and grasslands in semidesert mountains |  |
| *P. hieracioides* L. subsp. *hieracioides* |  | 2*n* = 10, 15 |  |  |
| PESK | IT, Abruzzo, Pescara, 6 m, 42º27.489’N, 14º12.596’E, col. M. Slovák, 22.6.2004 (SAV) |  | Xerothermous lowland to midaltitudinal rocky slopes and grasslands, ADH |  |
| CZ1/3 | CZ, Jihomoravský kraj county, Břeclav town, N edge of town, 166 m, 48°46′50′′N, 16°54′19′′E, col. M. Slovák and J. Kučera, 21.9.2011 (SAV) |  | Xerothermous lowland to midaltitudinal rocky slopes and grasslands, ADH |  |
| PH22/1 | TR, Kütahya province, Saphane Dağ, above Yumrutaş village, 1355 m, 39.0451415°N, 29.3222911°E, col. M. Slovák, J. Kučera, A. A. Dönmez, S. Yüzbaşioğlu, 4.7.2022 (SAV) |  | Xerothermous lowland to midaltitudinal rocky slopes and grasslands, ADH |  |
| *P. hieracioides* L. subsp. *hispidissima* (Bartl.) Slovák et Kučera |  | 2*n* = 10 |  |  |
| LOV1 | ME, Cetije, near Cetinje village, 485 m, 42°24′29′′N, 18°46′45′′E, col. M. Slovák, J. Kučera and A. Guttová 12.6.2011 (SAV) |  | Xerothermous calcareous rocky slopes in coastal mountain, ADH |  |
| PAK2 | HR, Ličko-senjska županija, Velika Paklenica valley, 30 m, 44°17′00′′N, 15°27′00′′E, col. M. Slovák, J. Kučera, A. Guttová, 9.6.2010 (SAV) |  | Xerothermous calcareous rocky slopes in coastal mountain, ADH |  |
| *P. hieracioides* subsp. *umbellata* (Schrank) Ces. |  | 2*n* = 10, 20 |  |  |
| AM3/1 | AM, Lori distr., Vanadzor town, above Pushkin pass, 40°55.166′N, 44°24.097′E, 2204 m, coll. J. Kučera and M. Slovák, 21.6.2019 (SAV) |  | Humid mountain grasslands, forest margins, open tall herb communities, ADH |  |
| MAD4 | SK, Žilinský kraj, Západné Tatry Mts., Mačie Diery, 900 m, 49° 15.525’N, 19° 40.234’E, col. M. Slovák, 2004 (SAV) |  | Humid mountain grasslands, forest margins, open tall herb communities, ADH |  |
| Gotl2 | SE, Öland, near Sandvik village, 7m, ca. 57°04′36′′N, 16°51′35′′E, col. P. Mereďa and I. Hodálová, 4.9.2012 (SAV) |  | Humid mountain grasslands, forest margins, open tall herb communities, ADH |  |
| *P. hispanica* (Willd.) P.D.Sell |  | 2n = 10, 20, 30 |  |  |
| MAF1 + MAF3 | MA, Souss-Massa Draa region, ca. 2 km S of the Tizi n′Tarkatine village, 1600 m, 29°44′33′′N,8°49′55′′W, col. M. Slovák, J. Kučera, A. Guttová and K. Olšavská, 2.5.2013 (SAV) |  | High mountain rocky slopes and grasslands, alpine meadows, ADH |  |
| MAAI6 | MA, Fes Boulemane region, S of Boulemane village, 1827 m, 33°20′26′′N, 4°42′49′′W, col. M. Slovák, J. Kučera, A. Guttová and K. Olšavská, 10.5.2013 (SAV) |  | High mountain rocky slopes and grasslands, alpine meadows, ADH |  |
| MAS1 | MA, Marrakesh Tensiftel Haouz region, above Igunane village, near Oukaimeden, 2245m, 31°14′16′′N, 7°49′05′′W, col. M. Slovák, J. Kučera, A. Guttová and K. Olšavská, 5.5.2013 (SAV) |  | High mountain rocky slopes and grasslands, alpine meadows, ADH |  |
| *P. japonica* Thunb. |  | 2*n* = 10, 20 |  |  |
| JP106/1 | JP, Akita pref., Kitaakita-gun, Tashiro-cho, Hirataki, 339 m, 40°22′23′′N, 140°26′20′′E, col. K. Marhold and J. Zozomová-Lihová, 29.6.2004 (SAV) |  | Humid mountain slopes, forest margins, open tall herb communities, ADH |  |
| *P. kotschyi* Boiss. |  | 2*n* = 10 |  |  |
| TRAB2 | TR, Kahramanmaraş province, Kilili village, 464 m, 37°27′47′′N, 36°53′25′′E, col. M. Slovák and J. Kučera, 24.5.2012 (SAV) |  | Xerothermous lowland, midaltitudinal rocky slopes and grasslands, ADH |  |
| TRZ2 + TR15 | TR, Osmanyie province, Taprakkale ruins, 123 m, 37°03′01′′N, 36°08′09′′E, col. M. Slovák and J. Kučera, 23.5.2012 (SAV) |  | Xerothermous lowland, midaltitudinal rocky slopes and grasslands, ADH |  |
| Half | TR, Şanliurfa province, Halfeti, 508 m, 37°14′28′′N, 37°52′31′′E, col. M. Slovák, 2004 (SAV) |  | Xerothermous lowland, midaltitudinal rocky slopes and grasslands, ADH |  |
| *P. longirostris* Sch. Bip. |  | 2*n* = 10 |  |  |
| IZ12/7 | IL, Southern district, W of Masada, 257 m, 31°19′22′′N, 35°19′26′′E, col. M. Slovák and J. Kučera, 4.4.2012 (SAV) |  | Wadi in deserts and semideserts mountains, ADH |  |
| IZ6/8 | IL, Southern district, SE of Arad town, 317 m, 31°12′32′′N, 35°15′07′′E, col. M. Slovák and J. Kučera, 2.4.2012 (SAV) |  | Wadi in deserts and semideserts mountains, ADH |  |
| IZ7/3 | IL, Southern district, Wadi Ashalim, near fort Tamar, 399 m, 31°01′32′′N, 35°14′37′′E, col. M. Slovák and J. Kučera, 2.4.2012 (SAV) |  | Wadi in deserts and semideserts mountains, ADH |  |
| *P. nuristanica* Bornm. |  | 2*n* = 10 |  |  |
| NUR7, NUR8 | KG, [Narynskaya oblasť], Fergana (Fergana Kyrka Toosu) [Mts.], 2800 m, ca. 40°52′29′′N, 74°04′59′′E, col. N.A., date. N.A. (SAV) |  | Humid mountain stony and rocky slopes, forest margins, open tall herb communities |  |
| *P. olympica* Boiss. |  | 2*n* = 10 |  |  |
| UD9 | TR, Bursa province, Uludag Mt., ca. 1800 m, 40.1101774°N, 29.1507717°E, col.: M. Slovák, J. Kučera, A.A. Dönmez and S. Yüzbaşioğlu, 3.7.2022 (SAV) |  | High mountain rocky slopes and alpine meadows |  |
| BAB2 | TR, Denizli province, Babadağ Mts., 1940 m, 37.7421747°N, 28.8693817°E, col.: M. Slovák, J. Kučera, A.A. Dönmez and S. Yüzbaşioğlu, 6.7.2022 (SAV) |  | High mountain rocky slopes and alpine meadows |  |
| *P. pauciflora* Willd. |  | 2*n* = 10 |  |  |
| PP6/6 | GR, Alistrati, Aggitis Canyon, 128 m, 41°01.443′N, 24°00.330′E, col. M. Slovák, J. Kučera and A. Guttová, 21.6.2010 (SAV) |  | Xerothermous lowland, mid altitudinal calcareous rocky slopes and grasslands |  |
| AKP6 | TR, Kütahya province, Simav, near Akpinar village, 39.1932029°N, 28.7393978°E, ca. 750 m, col.: M. Slovák, J. Kučera, A.A. Dönmez and S. Yüzbaşioğlu, 5.7.2022 (SAV) |  | Xerothermous lowland, mid altitudinal calcareous rocky slopes and grasslands |  |
| PP8/6 | MK, Municipality of Ohrid, Ohrid town, 703 m, 41°06′24′′N, 20°48′57′′E, col. M. Slovák, J. Kučera and A. Guttová, 22.6.2010 (SAV) |  | Xerothermous lowland, mid altitudinal calcareous rocky slopes and grasslands |  |
| *P. rhagadioloides*(L.) Desf. |  | 2*n* = 10 |  |  |
| IZ17/3 a IZ17/5 | IL, Northern district, near Tiberias town, -190 m, 32°48′13′′N, 35°31′46′′E, col. M. Slovák and J. Kučera, 5.4.2012 (SAV) |  | Xerothermous lowland, rocky slopes and grasslands, man made habitats, ADH |  |
| PR4/5 | TR, Balikesir province, near the Gökçeyazı, 300 m, 39°37.690′N, 27°35.763′E, col. M. Slovák, J. Kučera and A. Guttová, 18.6.2010 (SAV) |  | Xerothermous lowland, rocky slopes and grasslands, man made habitats, ADH |  |
| PP1/3 | GE, Island of Crete, districtu Xania, Theodoros, ca 70 m, 35º30′39′′N, 24º02′05.55′′E, col. D. Zelený, J. Danihelka and CH.F. Li (SAV) |  | Xerothermous lowland, rocky slopes and grasslands, man made habitats, ADH |  |
| TRR7 | TR, Antalya province, Azapkiri village, 3 m, 36°56.396′N, 30°55.495′E, col. M. Slovák and J. Kučera, 20.5.2012 (SAV) |  | Xerothermous lowland, rocky slopes and grasslands, man made habitats, ADH |  |
| *P. scaberrima* Ten. |  | 2*n* = 10 |  |  |
| CAST1 | IT, Calabria, Castrovillari, 386 m, 39°49′17′′N, 16°12′11′′E, col. M. Slovák, 3.7.2005 (SAV) |  | Lowland and midaltitudinal xerothermous and calcareous grasslands, ADH |  |
| LUNG4 | IT, Calabria, Lungro, 453 m, 39°43′28′′N, 16°09′07′′E, col. M. Slovák, 5.7.2005 (SAV) |  | Lowland and midaltitudinal xerothermous and calcareous grasslands, ADH |  |
| *P. scabra* subsp. *abyssinica* (Sch. Bip.) M. Smalla |  | 2*n* = 10 |  |  |
| P696 | SA, Makkah province, 30 km from Taif, 21°20′53′′N, 40°30′35′′E, col. G. Popov, 1.7.1971 (BM) |  | Sandy dunes in inland desert and semidesert |  |
| P782 | ER, Asmera town, behind Saint Michal church, 2350 m, O.Rydling 1192, 8.5.1988 (ETH 036837) |  | Sandy dunes in inland desert and semidesert |  |
| *P. scabra* subsp. scabra Forssk. |  | 2*n* = 10 |  |  |
| P712 | YE, [Ibb governorate], near Mashwara Mt., Odayn-Ibb pass, ca. 2400 m., [13°58'17.95"N, 44°6'10.85"E], leg. J.R.I. Wood 1657, 31.5.1977 (BM 81347) |  | High mountain humid rocky slopes and meadows |  |
| *P. sinuata* (Lam.) Lack |  | 2*n* = 10 |  |  |
| SIN4 | TN, Nabeul governorate, El Haouaria village, Ghar el Kebir, 37°03.451′N, 10°59.694′E, 18 m, leg. M. Slovák and A. Bérešová, 26.3.2014 (SAV) |  | Coastal sandy dunes |  |
| *^#^* TN1 | TN, Sousse governorate, Sousse, ca. 35°50′01′′N, 10°38′02′′E, col. F. Skhiri, A. El Assi and M. van Slageren, 1997 (K, SAV) |  | Coastal sandy dunes |  |
| *P. strigosa* M. Bieb. |  | 2*n* = 10 |  |  |
| AM1/3 | AM, Lori distr., near Shnogh village, 599 m, 41°08.412′N, 44°50.214′E, col. J. Kučera & M. Slovák, 21.6. 2019 (SAV) |  | Dry to humid high mountain rocky slopes |  |
| TRAA9 | TR, Kahramanmaraş province, near Türkoğlu town, 469 m, 37°20′37′′N, 36°49′37′′E, col. M. Slovák and J. Kučera, 23.5.2012 (SAV) |  | Dry to humid high mountain rocky slopes |  |
| LB1/2 | LB, Mount Lebanon Gov., above Maasser El Shouf village, 1424 m, 33°40.092′N, 35°40.829′E, col. J. Kučera & M. Slovák, 19.5.2019 (SAV) |  | Dry to humid high mountain rocky slopes |  |
| IR-PI1/2 | IR, Yazd province, Tezerjan village, 2160 m, 31°37′17′′N, 54°11′11′′E, col. M. Slovák, 14.5.2011 (SAV) |  | Dry to humid high mountain rocky slopes |  |
| *P. willkommii* (Willk.) Nyman |  | 2*n* = 10 |  |  |
| 2011a | ES, Huelva [province], Ayamonte [village], [37°12′52′′N, 7°24′15′′W], col. B. Valdés, 1.7.2004 (SAV) |  | Xerothermous rocky lowland grasslands |  |
| **Outgroups** |  |  |  |  |
| *Crepis biennis* L. | SK, Bratislavský kraj county, Bratislava city, Institute of Botany, 48.1728794°N, 17.0667344°E, col. M. Slovák, 10.8.2022 (SAV) | - | - |  |
| **Gundelia tournefortii* L. | IR, N. A., col. N. A., date N. A. (US 1252434) | - | - |  |
| *Helminthotheca echioides* (L.) Holub - HE1 | IT, Sicily, Rebottone, 680 m, 38°01′43′′N, 13°21′59′′E, col. M. Slovák, 17.6.2004 (SAV) | - | - |  |
| *Hypochaeris achyrophorus* L. - TN2014 | TN, [BenArousgovernorate], Hamman-Lif city, BoukornineMt., 93 m, 36°43′33′′N, 10°19′42′′E, col. M. Slovák and A. Guttová, 2014 (SAV) | - | - |  |
| *Hypochaeris radicata* L. - HR22/1 | SK, Bratislavský kraj county, Bratislava city, Institute of Botany, 48°10′22′′N, 17°04′00′′E, col. M. Slovák, 8.7.2014 (SAV) | - | - |  |
| ***Lactuca perennis* L. | IT, Piemont, Cuneo, M. Ristow, D. Lauterbach and B. Gemeinholzer MiRi 578/09, 12.7.2009 (B) | - | - |  |
| ***Lactuca serriola* L. | TR, Artvin province, Kafkasör'e çıkarken, 1072 m, Coskuncelebi and Güzel 141, 10.9.2013 (KTUB) | - | - |  |
| *Leontodon hispidus L. -* LH22/1 | SK, Bratislavský kraj county, Bratislava city, Institute of Botany, col. M. Slovák, 10.8.2022 (SAV) | - | - |  |
| *Leontodon tingitanus* (Boiss. et Reut.) Ball - 134858_9 | ES, (Cádiz [province]): Paloma Baja [village] N Punta Paloma, [36°03′40′′N, 5°42′54′′W], col. Gutermann 37432, date N.A. (WU) | - | - |  |
| ***Nabalus albus* (L.) Hook | US, Campbell County, Tennessee, Schilling, E. 3225, date N. A. (TENN) | - | - |  |
| **Prenanthes boottii* (DC.) D.Dietr. | US, Coos County, New Hampshire, col. H. Harries 172, date N. A. (TENN) | - | - | SRR9119046 |
| **Scolymus hispanicus* L. | ES, Andalucia, N. A., col. L.E.Watson, 6.1995 (MU) | - | - | SRR9119015 |
| *Scorzoneroides autumnalis* (L.) Moench - SA22/1 | SK, Bratislavský kraj county, Bratislava city, Institute of Botany, col. M. Slovák, 8.7.2014 (SAV) | - | - |  |
| ***Sonchus ustulatus subsp. maderensis* Aldridge | PT, Madeira, N. A., J. Suda, 2004 (PRC) | - | - |  |
| ***Sonchus pinnatus* Aiton | ES, Canary Islands, Tenerife, N. A., J. Suda, 2004 (PRC) | - | - |  |
| **Taraxacum kok-saghyz* L.E. Rodin | Greenhouse grown seed - USDA, W6 35156, J.R. Mandel 102, 27.8.2013 (GA) | - | - |  |
| **Tragopogon dubius* Scop. | US, Washington County, Oakesdale, col. D. Soltis 2674-4, date N. A. (WS) | - | - |  |

**REFERENCES**

**Astuti, G., Roma-Marzio, F., Peruzzi, L., 2015.** The genus *Picris* (Asteraceae) in southern Italy: contribution to its systematic knowledge. Phytotaxa 207, 106–114.

**Berjano, R., Talavera, M., Jiménez, F.J., Talavera, S., 2014.** *Picris cupuligera*, in: Marhold, K. (Ed.), IAPT/IOPB chromosome data 17. Taxon 63, 1148–1155.

**Galland, N., 1988**. Recherche sur l’origine de la ﬂore orophile du Maroc: étude caryologique et cytogéographique. Trav. Inst. Sci. Univ. Mohammed V, Sér. Bot. 35, 1–168.

**Holzapfel, S., 1994.** A revision of the genus Picris (Asteraceae, Lactuceae) s.l. in Australia. Willdenowia 24, 97–217.

**Holzapfel, S., 2015.** Picris, in: Wilson, A. (Ed.), Flora of Australia. Asteraceae 1, vol. 37, CSIRO Publishing, Canberra, pp. 143–155.

**Hong, D.Y., Zhang, S.Z.,** **1990**. Observations on chromosomes of some plants from western Sichuan. Cathaya 2, 191–197.

**Huang, J.M., Ge, C.J., 1988**. A study on the karyotype of Picris japonica. J. Shandong Univ. Traditional Chin. Med. 2, 57–58.

**Humphries, C.J., 1978.** Chromosome numbers of phanerogams from Morocco and Algeria. Bot. Not. 131, 391–404.

**Jones, K.E., Fér, T., Schmickl, R.E., Dikow, R.B., Funk, V.A., Herrando-Moraira, S., Johnston, P.R., Kilian, N., Siniscalchi, C.M., Susanna, A., Slovák, M., Thapa, R., Watson, L.E., 2019.** An empirical assessment of a single family-wide hybrid capture locus set at multiple evolutionary timescales in Asteraceae. Applications in Plant Sciences, 7(10), e11295.

**Kučera, J., Slovák, M., 2012.** Picris. in: Marhold, K., (ed.), IAPT/IOPB chromosome data 13. Taxon 61, 898.

**Kučera, J., Mártonfiová, L., Štubňová, E., Slovák, M., 2016.** Picris, in: Marhold, K., Kučera, J. (Eds.), IAPT/IOPB chromosome data 21. Taxon 65, 674–675.

**Lack, H.W., 1974**. Die Gattung Picris L. sensu lato im ostmediterran–westasiatischen Raum. Dissertationes der Universität Wien, 116 pp.

**Lack, H.W., 1977.** Picris sinuata (Lam.) Lack, comb. Nova (Asteraceae, Lactucaceae), eine verkannte Art aus Nordafrika. Willdenowia 8, 49–65.

**Lack, H.W., 1979.** The genus Picris (Asteraceae, Lactuceae) in Tropical Africa. Pl. Syst. Evol. 131, 35–52.

**Luque, T., Lifante, Z.D., 1991.** Chromosome numbers of plants collected during Iter Mediterraneum I in the SE of Spain. Bocconea 1, 303–364.

**Malallah, G.A., Brown, G., 1999**. Determination of chromosome number of Kuwaiti flora I. Cytologia 64, 181–196.

**Mandel, J.R., Dikow, R.B., Siniscalchi, C.M., Thapa, R., Watson, L.E. & Funk, V.A., 2019.** A fully resolved backbone phylogeny reveals numerous dispersals and explosive diversifications throughout the history of Asteraceae. Proceedings of the National Academy of Sciences U.S.A., 116(28), 14083–14088.

**Murray, B.G., Lange, P.J.D., 1999.** Contributions to a chromosome atlas of the New Zealand flora - 35. Miscellaneous families. New Zealand J. Bot. 37, 511–521.

**Nazarova, E.A., 1984.** Chromosome numbers in the Caucasian representatives of the families Asteraceae, Brassicaceae, Fabaceae, Limoniaceae. Bot. Zh. SSSR 69, 972–975.

**Oberprieler, C., Vogt, R., 1993.** Chromosome numbers of North African phanerogams. II. Willdenowia 23, 211–238.

**Peng, C.I., Hsu, C.C., 1978**. Chromosome numbers in Taiwan Compositae. Bot. Bull. Acad. Sin. 19, 53–66.

**Podlech, D., 1986.** Chromosome studies en Pflanzendes Saharo — Sindischen Trockengebletes MHH. Botanical Staatssamml München, 22, 5–20.

**Probatova, A., 2005**. Chromosome numbers of some dicotyledons of the flora of the Amur Region. Bot. Zh. SSSR 90, 779–792.

**Slovák, M., Šingliarová, B., Mráz, P., 2007.** Chromosome numbers and mode of reproduction in Picris hieracioides s.l. (Compositae) with notes on some other Picris taxa. Nord. J. Bot. 28, 238–244.

**Slovák, M., Vít, P., Urfus, T., Suda, J., 2009**. Complex pattern of genome size variation in a polymorphic member of the Asteraceae. J. Biogeogr. 36, 372–384.

**Slovák, M., Urfus, T., Vít, T., Marhold, K., 2009**. Balkan endemic Picris hispidissima (Compositae): morphology, DNA content and relationship to polymorphic P. hieracioides. Pl. Syst. Evol. 278, 187–201.

**Slovák, M., Kučera, J., Marhold, K., Zozomová-Lihová, J., 2012.** The morphological and genetic variation in the polymorphic species Picris hieracioides (Compositae, Lactuceae) in Europe strongly contrasts with traditional taxonomical concepts. Syst. Bot. 21, 258–278.

**Slovák, M., Kučera, J., Záveská, E. Vďačný, P.** **2014.** Dealing with discordant genetic signal caused by hybridisation, incomplete lineage sorting and paucity of primary nucleotide homologies: a case study of closely related members of the genus Picris subsection Hieracioides (Compositae). PLoS ONE 9, e104929.

**Smalla, M., 2000**. Studies in the Compositae of the Arabian Peninsula and Socotra 2000. The Hypochaeridinae (Lactuceae) in the Arabian Peninsula. Willdenowia 30, 315–339.

**Stepanov, N.V.**, 1994. Chromosome numbers of some higher plants taxa of the flora of Krasnoyarsk region. Bot. Zh. SSSR 79, 135–139.

**Talavera, S., 1979**. Numeros cromosomicos para la flora Espanola, 84-120. Lagascalia 9, 115–130.

**Table S2.** List of sampled populations used for karyological analyses.

| **Taxon/locality** | **Chromosome number** |
| --- | --- |
| ***P. campylocarpa* Boiss. et Heldr.** | |
| TR7 - TR, Antalya province, near Taskesigi village, 75 m, 36.752459°N, 31.624180°E, col. M. Slovák and J. Kučera, 12.6.2023 (SAV) | 2*n* = 10 |
| TR10 - TR, Antalya province, Yesiloz village, 75 m, 36.364348°N, 32.199932°E, col. M. Slovák and J. Kučera, 12.6.2023 (SAV) | 2*n* = 10 |
| TR13 - TR, Mersin province, Aydincik town, 200 m, 36.150064°N, 33.392241°E, col. M. Slovák and J. Kučera, 12.6.2023 (SAV) | 2*n* = 10 |
| ***P. cyprica* Lack** | |
| TR3 - TR, Antalya province, Kaputaş canyon, near Kalkan town, 5 m, 36°13′46′′N, 29°26′59′′E, col. M. Slovák and J. Kučera, 19.5.2012 (SAV) and col. M. Slovák and J. Kučera, 11.6.2023 (SAV) | 2*n* = 10 |
| TR8 - TR, Antalya province, near Murtiçi village, 524 m, 36°53′43′′N, 31°45′31′′E, col. M. Slovák and J. Kučera, 21.5.2012 (SAV) and col. M. Slovák and J. Kučera, 11.6.2023 (SAV) | 2*n* = 10 |
| ***P. kotschyi* Boiss*.*** | |
| TR, Osmanyie province, Taprakkale ruins, 123 m, 37°03′01′′N, 36°08′09′′E, col. M. Slovák and J. Kučera, 23.5.2012 (SAV) | 2*n* = 10 |

**Table S3.** List of characters used for ancestral state reconstructions.

| **Taxon** | **Longevity** | **Fruit morphology** | **Environment** |
| --- | --- | --- | --- |
| *P. amalecitana* | semelparous | heterocarpic | unpredictable |
| *P. campylocarpa* | semelparous | heterocarpic | unpredictable |
| *P. cyprica* | semelparous | homocarpic + heterocarpic | unpredictable |
| *P. galilaea* | semelparous | heterocarpic | unpredictable |
| *P. hieracioides* subsp*. hieracioides* | iteropar. + semelpar. | homocarpic | predictable + unpredictable |
| *P. hieracioides* subsp*. hispidissima* | semelparous | homocarpic | unpredictable |
| *P. hieracioides* subsp*. umbellata* | iteroparous | homocarpic | predictable |
| *P. japonica* | iteroparous | homocarpic | predictable |
| *P. kotschyi* | semelparous | heterocarpic | unpredictable |
| *P. longirostris* | semelparous | heterocarpic | unpredictable |
| *P. nuristanica* | iteroparous + semelparous | homocarpic | predictable |
| *P. olympica* | iteroparous | homocarpic | predictable |
| *P. pauciflora* | semelparous | homocarpic | unpredictable |
| *P. rhagadioloides* I | semelparous | heterocarpic | unpredictable |
| *P. rhagadioloides* II | semelparous | heterocarpic | unpredictable |
| *P. scaberrima* | iteroparous | homocarpic | unpredictable |
| *P. sinuata* | iteroparous + semelparous | heterocarpic | unpredictable |
| *P. strigosa* | iteroparous | homocarpic | predictable |
